# Supplementary material for: Brief self-guided digital intervention versus a comprehensive therapist-guided online cognitive behavioural therapy for atopic dermatitis: a trial protocol for a randomised non-inferiority trial
Source: BMJ Open. 2023 Feb 28;13(2):e068908. doi: 10.1136/bmjopen-2022-068908 (PMC9980367; doi:10.1136/bmjopen-2022-068908)
Supplement: Author's manuscript [file bmjopen-2022-068908.draft_revisions.pdf]

BMJ Open

BMJ Open is committed to open peer review. As part of this commitment we make the peer review history of every article we publish publicly available.

When an article is published we post the peer reviewers' comments and the authors' responses online. We also post the versions of the paper that were used during peer review. These are the versions that the peer review comments apply to.

The versions of the paper that follow are the versions that were submitted during the peer review process. They are not the versions of record or the final published versions. They should not be cited or distributed as the published version of this manuscript.

BMJ Open is an open access journal and the full, final, typeset and author-corrected version of record of the manuscript is available on our site with no access controls, subscription charges or pay-per-view fees (http://bmjopen.bmj.com).

If you have any questions on BMJ Open's open peer review process please email info.bmjopen@bmj.com

Brief Digital Self-care Intervention versus a Comprehensive Therapist-guided Online Cognitive Behavioral Therapy for Atopic Dermatitis: A Trial protocol for a Randomized Non-Inferiority Trial

Journal:	*BMJ Open*
Manuscript ID	bmjopen-2022-068908
Article Type:	Protocol
Date Submitted by the Author:	05-Oct-2022

Complete List of Authors:	Kern, Dorian; Karolinska Institutet, Department of Clinical Neuroscience; Stockholm Health Care Services, Department of Clinical Neuroscience<br>Ljótsson, Brjánn; Karolinska Institutet, Department of Clinical Neuroscience, Division of Psychology<br>Lönndahl, Louise; Karolinska Institutet, Department of Medicine Solna<br>Hedman-Lagerlöf, Erik ; Karolinska Institute<br>Bradley, M; Karolinska Institutet<br>Lindefors, Nils; Karolinska Institutet, Department of Clinical Neuroscience<br>Kraepelien, Martin; Karolinska Institutet

Keywords:	Eczema < DERMATOLOGY, Health economics < HEALTH SERVICES ADMINISTRATION & MANAGEMENT, Adult dermatology < DERMATOLOGY

SCHOLARONE™ Manuscripts

Trial protocol

Brief Digital Self-care Intervention versus a Comprehensive Therapist-guided Online Cognitive Behavioral Therapy for Atopic Dermatitis: A Trial protocol for a Randomized Non-Inferiority Trial

Dorian Kern, MSc[1,2]., Brjánn Ljótsson, PhD[2], Louise Lönndahl[3], MD, PhD., Erik Hedman-Lagerlöf[2], PhD[1], Maria Bradley, MD, PhD[3], Nils Lindefors MD, PhD[1], Martin Kraepelien, PhD[1,2]

[1]Centre for Psychiatry Research, Department of Clinical Neuroscience, Karolinska Institutet & Stockholm Health Care Services, Region Stockholm, Norra Stationsgatan 69, SE-113 64 Stockholm, Sweden

[2]Division of Psychology, Department of Clinical Neuroscience, Karolinska Institutet, Nobels väg 9 SE-171 65 Solna, Sweden

[3]Dermatology and Venereology Unit, Department of Medicine Solna, Karolinska Institutet, SE-171 77 Stockholm, Sweden

Correspondence: Dorian Kern; Centre for Psychiatry Research, Norra stationsgatan 69, 113 64, Stockholm; +467 23 24 32 35, dorian.kern@ki.se

Key words: Atopic dermatitis; eczema; pruritus; cognitive behavioral therapy; self-care

Word count: 3367

Version: 1.2

Date: 2022-28-09

For peer review only

Abstract

Introduction Our aim is to investigate whether a shortened digital self-care intervention is noninferior to, and cost-effective compared with, a comprehensive and therapist-guided CBT treatment for AD.

Methods and analysis This is a single-blind, randomized clinical non-inferiority trial at Karolinska Institutet, a medical university in Stockholm, Sweden. We will recruit 174 adult participants with AD through self-referral. Participants will be randomized 1:1 to the two experimental conditions. Participants randomized to guided care will receive internet-delivered cognitive behavior therapy for 12 weeks. Participants randomized to digital self-care will have access to this self-guided intervention for 12 weeks. At post-treatment (primary endpoint), non-inferiority will be tested and resource use will be compared between the two treatment groups. Cost-effectiveness will be explored at 1-year follow-up. Potential mediators will be investigated. Data will be analyzed intention-to-treat. We define non-inferiority as a 3-point difference on the primary outcome measure (Patient-oriented Eczema Measure). Recruitment is planned to start in late autumn 2022.

Ethics and dissemination This study is approved by the Swedish ethics authority (reg. no 2021-06704-01) and is preregistered at ClinicalTrials.gov. The study will be reported according to the Consolidated Standards of Reporting Trials statement for non-pharmacological trials. The results of the study will be published in peer-reviewed scientific journals and disseminated to patient organisations and media.

Trial registration: ClinicalTrials.gov identifier: NCT05517850, https://clinicaltrials.gov/ct2/show/NCT05517850

Strengths and limitations of this study

- First study comparing self-care and therapist guided cognitive behavioral therapy (CBT) for atopic dermatitis (AD).
- Includes a full health economics evaluation.
- Broad inclusion criteria will help generalize the results to a variety of AD cases.
- Depending on what time of the year the majority of participants eventually will be recruited, it may be difficult to control for seasonal changes in Sweden's temperate climate.

For peer review only

- Earlier similar studies indicate a risk of bias for the recruitment of female participants with an advanced education.

Background

Atopic dermatitis

Atopic dermatitis (AD) is the most prevalent of the inflammatory skin diseases. The primary symptoms of AD are itch, dry skin, and rash[1]. Treatment and prevention generally consist of emollient creams, topical corticosteroids, other local immunosuppressive creams such as calcineurin inhibitors. If topical treatment is ineffective, phototherapy and systemic immunomodulatory treatments are often given[2]. In addition to dermatological symptoms, people with AD have an increased risk of major depression and anxiety disorders[3, 4]. Furthermore, behavioral and emotional factors may affect AD symptoms. A common reaction to the itching sensation of the skin is the act of scratching, which, while temporarily relieving, can lead to damage to the skin barrier and an aggravated inflammation[5]. Furthermore, people with AD tend to scratch when they experience anxiety or depressed mood[6], possibly as a conditioned behavior. In addition, avoidance of itch-associated situations is frequently documented, which may lead to avoidance of activities that are otherwise appreciated or important to the patient, which can further affect mental health and quality of life[7] The impairment of quality of life by AD is comparable to asthma and type 1 diabetes[2]. The facts that AD can affect mental health, and that behavior can directly affect AD symptoms, make psychological treatment relevant.

Internet-delivered cognitive behavioral therapy

Internet cognitive-behavioral therapy (ICBT) is a psychological treatment administered over the Internet, otherwise similar to regular face-to-face CBT. ICBT usually includes therapist support via a secure digital communication, similar to e-mail. Studies have shown that a clinician spends the equivalent of about fifteen minutes per patient and week in ICBT[8]. ICBT has been suggested to improve cost-effectiveness as well as the availability of evidence-based treatment[9] and research reviews show that there are no consistent differences in effectiveness between conventional face-to-face CBT and

ICBT[10]. In Sweden, ICBT has been offered in regular care via the Internet Psychiatry Unit in Stockholm since 2007[11].

There is an unmet need for interventions for people with mild to moderate illness, where a comprehensive and therapist-guided ICBT program would be an unnecessarily intense level of care, and thus an inefficient use of resources. One solution is to use existing ICBT programs and simply remove the therapist-guidance, but previous research has suggested that this has negative impact on effectiveness[12]. However, there are indications that the amount and type of therapist contact are not decisive[13] and recent studies have compared therapist supported ICBT and self-guided ICBT and shown that treatment outcome, treatment implementation and treatment satisfaction do not differ between conditions if certain components are present in the self-guided intervention. These components include a stringent clinical process in the form of an assessment and a follow-up interview with a clinician and a high-quality interface[14, 15].

Internet-delivered cognitive behavioral therapy for atopic dermatitis

An ICBT protocol has been evaluated for AD. The main components were symptom diary, mindfulness, and exposure to avoided sensations and situations, with prevention of unhelpful responses. The treatment showed promising preliminary effects in a face-to-face pilot study, which subsequently was confirmed in a randomized controlled trial (RCT)[16, 17]. A digital self-care intervention has been preliminarily evaluated for people with AD, with promising effects[18]. Digital self-care was based on the ICBT intervention with comparable components. Thus, there is support that CBT can be effective in improving mental health and reducing symptoms of AD, although additional studies are needed. A comparison between the ICBT and reworked digital self-care variants could further contribute to the literature and the results will have implications for implementation.

This is a study protocol of a single-blind non-inferiority RCT, aimed at evaluating the effects of a self-guided CBT program available via the internet (digital self-care) for people with AD, in relation to a previously evaluated therapist-guided internet-delivered CBT program (guided care)[18]. The main hypothesis is that digital self-care will be non-inferior to guided care in reducing self-rated eczemic symptoms, as

For peer review only

measured by the Patient-oriented eczema measure (POEM)[19]. Secondary purposes are to evaluate use of resources and cost-effectiveness of digital self-care compared to guided care, and to investigate potential mediators of symptom improvement.

Hypotheses

Primary hypothesis: Digital self-care will be non-inferior to ICBT, according to the predefined non-inferiority margin of 3 points, as measured by the primary outcome (POEM).

Secondary hypothesis 1: Digital self-care will be associated with significantly less staff costs than ICBT, in the form of time spent in hours by a licensed psychologist.

Secondary hypothesis 2: Digital self-care will be cost-effective compared to ICBT, assessed at one-year follow-up.

Secondary hypothesis 3: Effects on the outcome will be mediated by the amount of time spent by participants on main treatment components.

Study design

The present study is a noninferiority RCT for adults with AD. Participants randomized to guided care will receive ICBT via the internet, guided by a therapist for 12 weeks. Participants randomized to digital self-care get access to a self-guided CBT-based intervention for 12 weeks. Condition allocation will be 50/50.

The primary end point is at 12 weeks, (i.e., post-treatment). The study will also include a six-month and a one-year follow-up. 1 year after treatment conclusion, long-term effects will be evaluated. An overview of the trial design can be seen in Figure 1.

The study is approved by the Swedish ethics authority (reg. no 2021-06704-01) and is be preregistered at ClinicalTrials.gov. The participants will receive written and verbal information about the study. The participants will digitally sign an informed consent in a secure platform.

For peer review only

Participants

Participants will be recruited via advertisements in social media and with the help of relevant patient associations' mailing lists and websites. Interested individuals with self-reported AD diagnosis may participate following their written consent to the study. In order to minimize false diagnoses, participants will disclose at which point in time they received their AD diagnosis, and if they can recall, at what clinic and by which physician. They will then receive login information to access a secure digital platform, where data from self-assessment scales will be registered. Participation in the study does not involve any change in their regular treatment or medical self-care. Participants will be evaluated by a licensed psychologist in a clinical interview, assessing suitability and motivation.

Inclusion criteria: At least 18 years of age with self-reported AD diagnosis. No new types of medications introduced for six months prior, with no intention of future change. Participants need to be able to understand written and spoken Swedish, have access to the internet and be able to receive telephone calls and text messages.

Exclusion criteria: Other disease or condition that has immediate treatment priority over AD. This could be an ongoing demanding treatment of a severe somatic or psychiatric condition. No specific diagnoses are inevitably a cause for exclusion, as potential participants will rather be evaluated on a case-to-case basis.

Setting

The trial is conducted at Karolinska Institutet, a medical university in Stockholm, Sweden. Dermatologists involved in the study are also associated with the dermatology clinic at Karolinska University Hospital, in Stockholm, Sweden. Assessment will be made by telephone, and both study arms are entirely conducted online. Participants will be recruited from all parts of Sweden.

Power calculation

Based on data from Hedman-Lagerlöf and colleagues[17] and Kern and colleagues[18] we expect an in-group effect of about d=0.85 for both interventions. A consultant statistician performed a power calculation. The required sample size was computed

For peer review only

based on boot-strapped (5000 simulations) reruns of weekly measured data obtained in the therapist-guided RCT[17]. The results indicated that a sample size of 174 would be required for 90% power, α=.05, given a non-inferiority margin of 3 points on the POEM scale and accounting for 10% missing data.

Planned interventions

Guided care

This condition consists of 10 modules and lasts 12 weeks. An introduction provides information about CBT and AD. Participant register itching and scratching behaviors, with the aim to increase awareness of these behaviors. Gradually, the participants are introduced to the proposed active treatment components: mindfulness training, and exposure with response prevention. Mindfulness is trained throughout the intervention. The purpose of mindfulness is to increase awareness of emotional and behavioral reaction patterns, and to increase tolerance of aversive inner experiences[20]. Additionally, mindfulness training may improve stress tolerance. Participants are also trained in self-compassion, to increase understanding of one's own reactions and to increase the ability to handle difficult situations in the treatment and life[21]. After practicing these fundamental skills, exposure with response prevention is introduced in week 4. Exposure is a controlled way to gradually approach situations that evoke strong emotions or somatic reactions. The purpose is to gain knowledge and experience in how this approach can change one's perception of unwanted situations, emotions, or somatic reactions[22]. The assumption is that skills learned from mindfulness practice and self-compassion training helps the patient to conduct exposure exercises. Response prevention means to reduce scratching in these problematic situations in order to reduce itch in the long term. Through this whole process, the participant is guided by a therapist. The therapist may be contacted through a secure internet platform at any time and responds within 48 hours. Participants reads the education material, construct exercises to perform, describes their progress in homework assignments, and receives feedback from their therapist.

For peer review only

Digital self-care

Similar to guided care, the participants will access a digital platform, read information, and perform exercises in their everyday life. As with guided care, the main treatment components are mindfulness and exposure with response prevention, with a specific focus on itch. Participants will be able to practice mindfulness with the help of instructions and examples. Because it may be challenging to construct relevant exposure exercises without the help of a therapist, participants will receive detailed instructions and guidance, and get to choose exposure exercises from a long list of examples. They will also be free to create their own relevant exposure situations within the intervention. Digital self-care consists of 8 modules. The only active interventions, mindfulness and exposure are introduced week 1 and 2, respectively. From week 3-7, no new interventions are introduced and participants are advised to continue practicing the interventions, but there is a new theme of education each week, to act as inspiration for exercises. The last 4 weeks, the participant is merely advised to continue practice.

Differences between study conditions

The main differences between the conditions are that digital self-care a) has contact with a clinician at only two points in time: pre intervention and after eight weeks plus the addition of automated messages, whereas guided care has continuous contact throughout the treatment via text messages on a secure platform, and that b) the treatment text is significantly shortened. Guided care will also have an evaluation from a clinician via telephone at two points in time. Furthermore, digital self-care uses a mobile optimized interface, and have improvements in design aimed to decrease the need of guidance.

Therapists

Only the guided group will have therapist contact. The self-guided group will be managed by the study coordinator. The therapists are licensed psychologists specialized in CBT. All therapists will participate in an introductory workshop before treating in the study. The project coordinator will supervise the therapists regularly and on-demand.

For peer review only

Randomization/blinding

Participants will be blinded to condition. They will be generally informed that they will be randomized to “either one of two types of self-help interventions based on CBT”, not knowing any difference between the conditions. After baseline measurements, included participants will be allocated randomly (1:1) to therapist-guided or self-guided treatment in consecutive even-numbered blocks. Randomization will be based on a true random number service and conducted by a person blinded to the participants, who is not otherwise involved with the study, using a true random number generator (http://www. random.org). It is impossible for the therapists performing the inclusion assessment to anticipate participant allocation.

Measurements

The outcome measures are based on those used previously by Hedman-Lagerlöf and colleagues[17] and Kern and colleagues[18], for the evaluation of CBT interventions for AD.

Primary outcome measure: Patient Oriented Eczema Measure (POEM)[17] measures the severity of symptoms of atopic dermatitis, which has a range of 0 to 28. The POEM has excellent psychometric properties and is widely used[23]. In this study, in order to be conservative, response is operationalized as a POEM score reduction 4 points or more, and the non-inferiority margin is operationalized as 3 points[23].

Secondary outcome measures: Peak pruritus numerical rating scale (NRS)[24], measuring itching sensations; Perceived Stress Scale (PSS)[25] is used to measure general experience of stress; Patient Health Questionnaire-9 (PHQ-9)[26] is used to measure depressive symptoms; Dermatological Life Quality Index (DLQI)[27] measures AD-specific quality of life; Insomnia severity index (ISI)[28] measures severity of sleep problems and possible insomnia; Client Satisfaction Questionnaire (CSQ-8)[29] are used to measure how satisfied participants are with the intervention. We will also ask participants to report if they experience adverse events associated with the treatment. In addition to this, we will use the Credibility / Expectancy Scale (CES)[30] to measure credibility. Each week, the participants will be asked to estimate the time spent in the intervention. Resource use measured by the time the clinician spends on the different tasks associated with administering the intervention, doing interviews, and giving support. Euro-QOL 5 dimensions (EQ-5D)[31] will be used to assess quality of life from

For peer review only

a health economics perspective. Treatment Inventory of Costs in Psychiatric Patients (TIC-P)[32] will be used to assess health-care costs.

Outcomes at all scales will be compared with available published results regarding other Internet-based interventions for each scale.

Measurement points

Screening: PHQ-9, POEM

Pre-intervention: POEM (primary outcome measure), NPR, DLQI, PHQ-9, PSS. TIC-P, EQ-5D

Post-intervention (primary endpoint): POEM (primary outcome measure), NPR, DLQI, PHQ-9, PSS. Survey only: CSQ-8, Adverse events TIC-P, EQ-5D

After two weeks**:** CES

Intermediate measurement: POEM (primary outcome measure), NPR, DLQI, PHQ-9, PSS, TCS, TIC-P, EQ-5D

Weekly measurement: POEM, NPR, PHQ-9, PSS, estimate time spent on exercises

Six-month follow-up: POEM (primary outcome measure), NPR, DLQI, PHQ-9, PSS TIC-P, EQ-5D

One year follow-up: POEM (primary outcome measure), NPR, DLQI, PHQ-9, PSS, TIC-P, EQ-5D

There are no planned interim analyses. There are no predefined criteria for trial termination.

For peer review only

Safety considerations

Participants' regular healthcare is unaffected by either of the study conditions. Possible benefits for patients with increased ability to manage their disease are presumed to justify any disadvantages in the form of time consumption and temporary discomfort. Adverse treatment effects will be examined both during the intervention and after the total twelve weeks of the intervention. If the disease worsens, the participants will be encouraged to seek help from their regular specialist clinic or primary health care center. All communication and data storage take place on an encrypted and secure digital platform and is in accordance with the Karolinska Institutet's regulations and the EU General Data Protection Regulation. In case of any serious harm, the standard patient's insurance for Government financed health care will be applicable to every participant.

Statistical analysis

Non-inferiority margin

Earlier research[22] has defined clinically significant improvement on the main outcome measure to be 3,4 points, which is the basis for our non-inferiority margin of 3 points.

Non-inferiority

Change over time will be modelled using mixed-effects linear regression with a random intercept and slope (time). The main outcome POEM will be analyzed by the regression on time (baseline to end point), group (digital self-care vs guided care), and the interaction of time and group. We will consider digital care to be non-inferior to therapist-guided if the upper bound of the 1-sided 95% confidence interval for the time × group interaction ($\alpha = .05$) is within the a-priori non-inferiority margin of 3 points on the POEM[33]. In addition, we will investigate non-inferiority in a sensitivity analysis per-protocol framework with data from patients who will have initiated module 4 or further in either of the study arms. As for interpretation of results, if the two-sided 95% confidence interval does not contain zero, this will be viewed as indicative of superiority of the corresponding treatment. We will estimate Cohen's *d* by

For peer review only

dividing the time × group coefficient by the pooled observed baseline standard deviation. Significant missing data will be analyzed using multiple imputation.

Resource utilization

At the primary endpoint, resource utilization will be preliminary assessed by measuring therapist time for each treatment condition and compared with a typical salary for a clinical psychologist employed in Stockholm, Sweden.

Cost-effectiveness

At one-year follow-up, a cost-effectiveness analysis will be performed from a-healthcare system perspective, based on recorded therapist time, as well as from a societal perspective. The incremental cost-effectiveness ratio will be estimated at post-treatment and at follow-up. We will also use the TIC-P to collect economic cost data[31], together with the EQ-5D[32]. The costs will be assessed in Swedish krona and converted into US dollar using 2023 as the reference year. The costs of the self-guided and therapist-guided treatments will be modelled as a function of therapist time using the same healthcare index to determine tariffs for licensed psychologists. According to our hypothesis, there should be a significant difference in treatment costs but there should not be any significant difference in societal costs. We will calculate quality-adjusted life-years using the area under the curve approach[33], use regression analyses to analyze costs[34], and use nonparametric bootstrapping with 5000 iterations, where differences in costs and outcomes will be paired. The incremental cost-effectiveness ratio will be used as a cost-effectiveness estimate[35].

For peer review only

Mediation

We are interested in the mediating effect of time spent on mindfulness and exposure exercises on the outcome. Because we have a non-inferiority hypothesis, we will not perform traditional mediation analysis where the mediator is proposed to account for between-group differences. Instead, we will do within-group process analysis to investigate if treatment activity predicts simultaneous and/or subsequent change in POEM during treatment[36]. The exact analytic model will be determined based on the nature of data that we are able to collect on treatment activity. We will also explore potential moderated mediation, i.e., if mediators differ between the two types of

treatment. We also intend to analyze self-rated pruritus, perceived stress, and depressed mood as potential mediators. *

Data

All data will be stored at a secure digital platform, to which only study personnel have access. Measurements from the participants will be submitted in the platform itself. Because all data is submitted and stored in the same digital platform, we will not use a data monitoring committee. The final trial data set will be primarily handled by the first, second and final author. However, all authors will be granted access to the data set if necessary. We have no plans to make an individual-level data set or any statistical code public. Data will be available upon reasonable request to the corresponding author.

Patient and public involvement

We have used input from participants in the feasibility trial[18], on which we based intervention improvements in the present study. We also considered feedback from a patient representative, who was not a trial participant. In the current trial, no participants were involved in decisions about study design, outcome measures, or research question. A patient organization for AD, as well as one for disorders related to asthma and allergies (Atopikerna and Astma & Allergiförbundet) will be involved in the recruitment of participants by informing their members about the study. We plan to disseminate the results of the research to study participants and to the aforementioned patient organizations.

Ethics and Dissemination

The study will be carried out according to the predefined standards of this trial protocol, the Declaration of Helsinki and Good Clinical Practice. This study will be reported according to the CONSORT statement for non-pharmacological trials[37]. The interventions that will be administered have deemed safe by consultant dermatologists and have been previously evaluated without serious adverse events[17,18]. This study will use the Vancouver criteria for authorship. The results of this study will be published in peer-reviewed scientific journals, be presented to the scientific community

For peer review only

at conferences. The results will also be communicated to study participants, patient organizations and clinicians.

Trial Status

We expect to start recruitment in November 2022. Any important changes to the trial protocol will be reviewed by the Swedish Ethical Review Authority and reported at clinicaltrials.gov.

Conclusions

The present study could be an important step towards the development, evaluation, and implementation of psychological self-care for patients with AD, which could potentially increase access to cost-effective evidence-based psychological interventions. If self-guided treatment were non-inferior (within predefined limits) and cost-effective, this could be implemented with relative ease, and therefore help dermatological healthcare to meet the great demand for evidence-based interventions in the dermatological field and help to improve symptoms of AD in an innovative way.

Acknowledgement

Author contributions

Dorian Kern: Study design, intervention design, drafting of manuscript, planning of statistical analysis. Brjánn Ljótsson: Study design, intervention design, planning of statistical analysis. Louise Lönndahl: Contributions to intervention and manuscript; Maria Bradley: Study concept and contributions to manuscript. Erik Hedman-Lagerlöf: Study concept, contributions to manuscript and planning of statistical analysis; Nils Lindefors: Study concept, study design, contributions to manuscript. Martin Kraepelien: Study concept, contributions to manuscript, intervention design, study design, planning of statistical analysis, principal investigator. All authors: Critical review and revision of manuscript.

For peer review only

Funding/support

This study will be funded in part by the Swedish Ministry of Health and Social affairs. The funder has no role in the design and conduct of the study and will have no influence of data collection, management, analysis, interpretation. The funder will have no part in preparation, review or approval of the manuscript, or the decision to submit the manuscript for publication.

Declaration of conflict of interest

Dr Ljótsson reported being a shareholder of DahliaQomit AB and holding copyright for a cognitive behavioral treatment manual for irritable bowel syndrome, with royalties paid from Pear Therapeutic, Inc, outside of submitted work. Dr Lönndahl reported receiving a personal fee from LEO Pharma and Sanofi Genzyme outside of submitted work. Dr Hedman-Lagerlöf reported being a shareholder of DahliaQomit AB and holding copyright for a cognitive behavioral treatment manual for irritable bowel syndrome, with royalties paid from Pear Therapeutic, Inc, outside of submitted work. No other disclosures were reported.

For peer review

References

1. Drucker AM, Wang AR, Li WQ, et al. The Burden of Atopic Dermatitis: Summary of a Report for the National Eczema Association. *J Invest Dermatol*. 2017;137(1):26-30. doi:10.1016/j.jid.2016.07.012
2. Weidinger S & Novak N. Atopic dermatitis. *Lancet*. 2016;387(10023):1109-1122. doi:10.1016/S0140-6736(15)00149-X
3. Yaghmaie P, Koudelka CW, Simpson EL. Mental health comorbidity in patients with atopic dermatitis. *J Allergy Clin Immunol.* 2013; 131(2): 428-33
4. Sandhu JK et al, Wu KK, Bui T-L, Armstrong AW. Association Between Atopic Dermatitis and Suicidality: A Systematic Review and Meta-analysis. *JAMA Dermatology*. 2019; 155(2): 178-87.
5. Wahlgren CF. Itch and atopic dermatitis: an overview. *J Dermatol.* 1999; 26(11): 770-9

6. Daunton A, Bridgett C, Goulding JM. Habit reversal for refractory atopic dermatitis: a review. *Br J Dermatol.* 2016; 174(3): 657-9.
7. Verhoeven L, Kraaimaat F, Duller P, et al. Cognitive, behavioral, and physiological reactivity to chronic itching: analogies to chronic pain. *International journal of behavioral medicine.* 2006; 13(3): 237-43.
8. Hedman E, Ljótsson B, Kaldo V, et al. Effectiveness of Internet-based cognitive behaviour therapy for depression in routine psychiatric care. *J Affect Disord*. 2014;155:49-58. doi:10.1016/j.jad.2013.10.023
9. Cavanagh K. Geographic inequity in the availability of cognitive behavioural therapy in England and Wales: a 10-year update. *Behav Cogn Psychother*. 2014;42(4):497-501. doi:10.1017/S1352465813000568
10. Carlbring P, Andersson G, Cuijpers P, et al. Internet-based vs. face-to-face cognitive behavior therapy for psychiatric and somatic disorders: an updated systematic review and meta-analysis. *Cogn Behav Ther*. 2018;47(1):1-18. doi:10.1080/16506073.2017.1401115
11. Lindefors N, El Alaoui S, Svanborg C. Internet-KBT ger effektiv behandling - Signifikant hållbar förbättring vid depression, paniksyndrom och socialt ångestsyndrom [Internet CBT provides effective treatment]. *Läkartidningen*. 2017;114:D77R. Published 2017 Jan 24.
12. Cuijpers P, Donker T, van Straten A, et al.. Is guided self-help as effective as face-to-face psychotherapy for depression and anxiety disorders? A systematic review and meta-analysis of comparative outcome studies. *Psychol Med*. 2010;40(12):1943-1957. doi:10.1017/S0033291710000772
13. Hirai M, Clum GA. A meta-analytic study of self-help interventions for anxiety problems. *Behav Ther*. 2006;37(2):99-111. doi:10.1016/j.beth.2005.05.002
14. Dear BF, Staples LG, Terides MD, et al. Transdiagnostic versus disorder-specific and clinician-guided versus self-guided internet-delivered treatment for Social Anxiety Disorder and comorbid disorders: A randomized controlled trial. *J Anxiety Disord*. 2016;42:30-44. doi:10.1016/j.janxdis.2016.05.004
15. Titov N, Dear BF, Staples LG, et al. Disorder-specific versus transdiagnostic and clinician-guided versus self-guided treatment for major depressive disorder and comorbid anxiety disorders: A randomized controlled trial. *J Anxiety Disord*. 2015;35:88-102. doi:10.1016/j.janxdis.2015.08.002

16. Hedman-Lagerlöf E, Bergman A, Lindefors N, et al.. Exposure-based cognitive behavior therapy for atopic dermatitis: an open trial. *Cogn Behav Ther*. 2019;48(4):300-310. doi:10.1080/16506073.2018.1504320
17. Hedman-Lagerlöf E, Fust J, Axelsson E, et al. Internet-Delivered Cognitive Behavior Therapy for Atopic Dermatitis: A Randomized Clinical Trial. *JAMA Dermatol*. 2021;157(7):796-804. doi:10.1001/jamadermatol.2021.1450
18. 2022;10.1001/jamadermatol.2022.3434. doi:10.1001/jamadermatol.2022.3434
19. Charman CR, Venn AJ, Williams HC. The Patient-Oriented Eczema Measure: development and initial validation of a new tool for measuring atopic eczema severity from the patients' perspective. *Arch Dermatol* 2004; 140:1513–19.
20. Hayes SC, Luoma JB, Bond FW, et al,. Acceptance and commitment therapy: model, processes and outcomes. *Behav Res Ther*. 2006;44(1):1-25. doi:10.1016/j.brat.2005.06.006
21. Clarke EN, Thompson AR, Norman P. Depression in people with skin conditions: The effects of disgust and self-compassion. *Br J Health Psychol*. 2020;25(3):540-557. doi:10.1111/bjhp.12421
22. Sewart AR, Craske MG. Inhibitory learning. In: Abramowitz JS, Blakey SM, editors. Clinical handbook of fear and anxiety: Maintenance processes and treatment mechanisms. Washington, DC, USA: American Psychological Association; 2020:265-285.
23. Schram ME, Spuls PI, Leeflang MM, et al. EASI, (objective) SCORAD and POEM for atopic eczema: responsiveness and minimal clinically important difference. *Allergy*. 2012;67(1):99-106. doi:10.1111/j.1398-9995.2011.02719.x
24. Yosipovitch G, Reaney M, Mastey V, et al. Peak Pruritus Numerical Rating Scale: psychometric validation and responder definition for assessing itch in moderate-to-severe atopic dermatitis. *Br J Dermatol*. 2019;181(4):761-769. doi:10.1111/bjd.17744
25. Cohen S, Kamarck T, Mermelstein R. A global measure of perceived stress. *J Health Soc Behav*. 1983;24(4):385-396.
26. Kroenke K, Spitzer RL, Williams JB. The PHQ-9: validity of a brief depression severity measure. *J Gen Intern Med.* 2001; 16(9): 606-13.
27. Finlay AY, Khan GK. Dermatology Life Quality Index (DLQI)--a simple practical measure for routine clinical use. *Clin Exp Dermatol.* 1994; 19(3): 210-6.

For peer review only - http://bmjopen.bmj.com/site/about/guidelines.xhtml

28. Morin, C.M. Insomnia: Psychological Assessment and Management. Guilford, New York; 1993
29. Attkisson CC, Zwick R. The client satisfaction questionnaire. Psychometric properties and correlations with service utilization and psychotherapy outcome. *Eval Program Plann*. 1982;5(3):233-237. doi:10.1016/0149-7189(82)90074-x
30. Devilly GJ, Borkovec TD. Psychometric properties of the credibility/expectancy questionnaire. *J Behav Ther Exp Psychiatry*. 2000;31(2):73-86. doi:10.1016/s0005-7916(00)00012-4
31. Bouwmans C, De Jong K, Timman R, et al. . Feasibility, reliability and validity of a questionnaire on healthcare consumption and productivity loss in patients with a psychiatric disorder (TiC-P). BMC Health Serv Res. 2013;13(1):217. doi:10.1186/1472-6963-13-217 - DOI - PMC - PubMed
32. Rabin R, de Charro F. EQ-5D: a measure of health status from the EuroQol Group. *Ann Med*. 2001;33(5):337-343. doi:10.3109/07853890109002087
33. Walker J. Non-inferiority statistics and equivalence studies. *BJA Educ*. 2019;19(8):267-271. doi:10.1016/j.bjae.2019.03.004
34. Matthews JNS, Altman DG, Campbell MJ, et al. Analysis of serial measurements in medical research. BMJ. 1990;300(6719):230-235. doi:10.1136/bmj.300.6719.230 - DOI - PMC - PubMed
35. Manning WG, Mullahy J. Estimating log models: to transform or not to transform?. *J Health Econ*. 2001;20(4):461-494. doi:10.1016/s0167-6296(01)00086-8
36. H. Hesser. Modeling individual differences in randomized experiments using growth models: recommendations for design, statistical analysis and reporting of results of internet interventions Internet Interv. 2015, 110-120, 10.1016/j.invent.2015.02.003
37. Schulz KF , Altman DG , Moher D . CONSORT 2010 Statement: updated guidelines for reporting parallel group randomised trials. *BMC Med* 2010;8:18.doi:10.1186/1741-7015-8-18

Figure 1.

CONSORT-flow diagram. CBT, cognitive–behavioral therapy; CONSORT, Consolidated Standards of Reporting Trials

For peer review only

Figure 1 CONSORT 2010 Flow Diagram

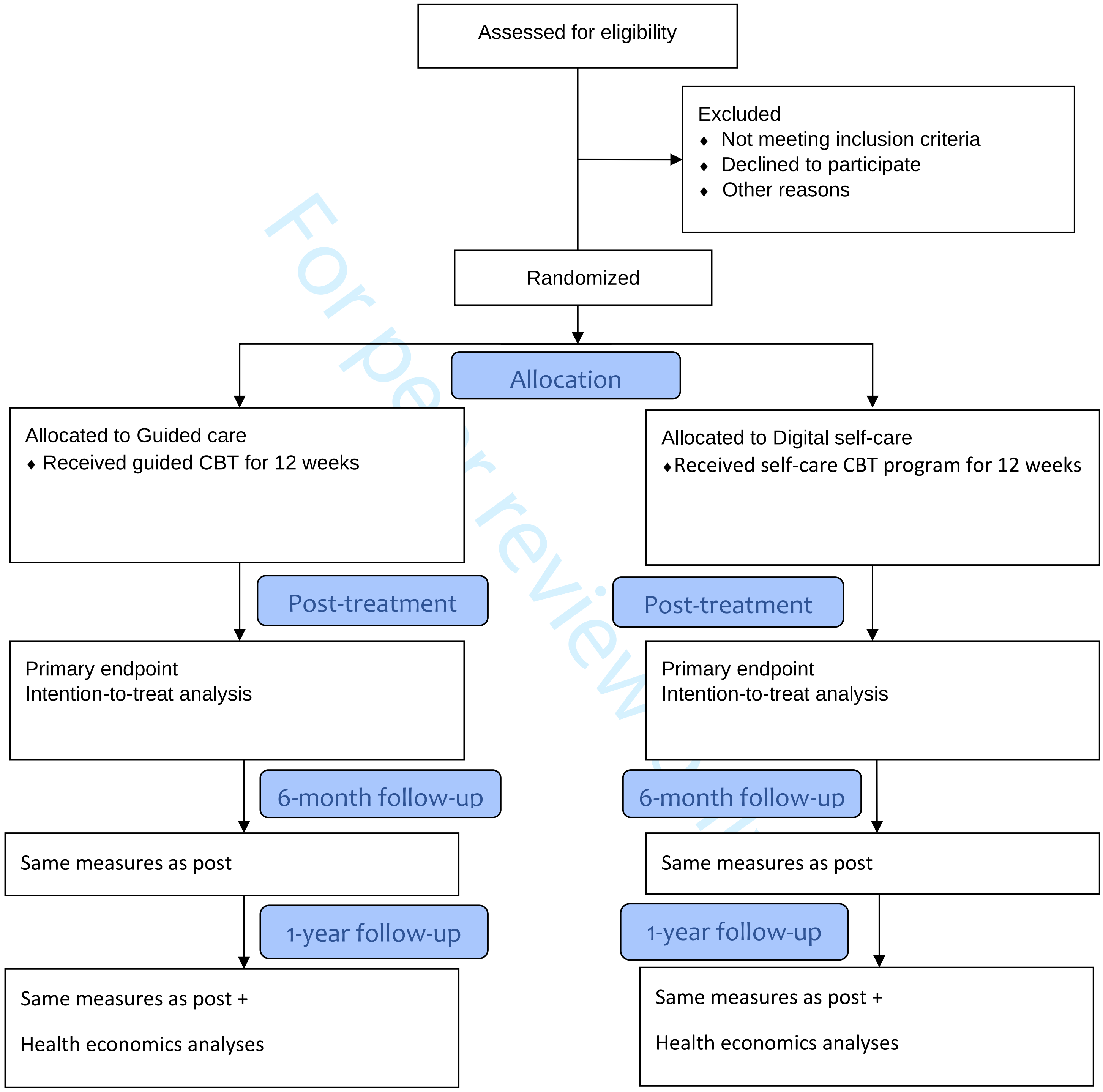

For peer review only

STANDARD PROTOCOL ITEMS: RECOMMENDATIONS FOR INTERVENTIONAL TRIALS

SPIRIT 2013 Checklist: Recommended items to address in a clinical trial protocol and related documents*

Section/item	Item No	Description	Addressed on page number
**Administrative information**			
Title	1	Descriptive title identifying the study design, population, interventions, and, if applicable, trial acronym	1
Trial registration	2a	Trial identifier and registry name. If not yet registered, name of intended registry	1
	2b	All items from the World Health Organization Trial Registration Data Set	1
Protocol version	3	Date and version identifier	1
Funding	4	Sources and types of financial, material, and other support	14-15
Roles and responsibilities	5a	Names, affiliations, and roles of protocol contributors	1, 14
	5b	Name and contact information for the trial sponsor	N/A (non-pharma. trial)
	5c	Role of study sponsor and funders, if any, in study design; collection, management, analysis, and interpretation of data; writing of the report; and the decision to submit the report for publication, including whether they will have ultimate authority over any of these activities	14-15
	5d	Composition, roles, and responsibilities of the coordinating centre, steering committee, endpoint adjudication committee, data management team, and other individuals or groups overseeing the trial, if applicable (see Item 21a for data monitoring committee)	N/A

For peer review only

**Introduction**			
Background and rationale	6a	Description of research question and justification for undertaking the trial, including summary of relevant studies (published and unpublished) examining benefits and harms for each intervention	3-4
	6b	Explanation for choice of comparators	6-7
Objectives	7	Specific objectives or hypotheses	5
Trial design	8	Description of trial design including type of trial (eg, parallel group, crossover, factorial, single group), allocation ratio, and framework (eg, superiority, equivalence, noninferiority, exploratory)	5
**Methods: Participants, interventions, and outcomes**			
Study setting	9	Description of study settings (eg, community clinic, academic hospital) and list of countries where data will be collected. Reference to where list of study sites can be obtained	6
Eligibility criteria	10	Inclusion and exclusion criteria for participants. If applicable, eligibility criteria for study centres and individuals who will perform the interventions (eg, surgeons, psychotherapists)	6
Interventions	11a	Interventions for each group with sufficient detail to allow replication, including how and when they will be administered	7-8
	11b	Criteria for discontinuing or modifying allocated interventions for a given trial participant (eg, drug dose change in response to harms, participant request, or improving/worsening disease)	N/A
	11c	Strategies to improve adherence to intervention protocols, and any procedures for monitoring adherence (eg, drug tablet return, laboratory tests)	8
	11d	Relevant concomitant care and interventions that are permitted or prohibited during the trial	7
Outcomes	12	Primary, secondary, and other outcomes, including the specific measurement variable (eg, systolic blood pressure), analysis metric (eg, change from baseline, final value, time to event), method of aggregation (eg, median, proportion), and time point for each outcome. Explanation of the clinical relevance of chosen efficacy and harm outcomes is strongly recommended	9-10

For peer review only - http://bmjopen.bmj.com/site/about/guidelines.xhtml

Participant timeline	13	Time schedule of enrolment, interventions (including any run-ins and washouts), assessments, and visits for participants. A schematic diagram is highly recommended (see Figure)	Diagram available
Sample size	14	Estimated number of participants needed to achieve study objectives and how it was determined, including clinical and statistical assumptions supporting any sample size calculations	6-7
Recruitment	15	Strategies for achieving adequate participant enrolment to reach target sample size	6
**Methods: Assignment of interventions (for controlled trials)**			
Allocation:			
Sequence generation	16a	Method of generating the allocation sequence (eg, computer-generated random numbers), and list of any factors for stratification. To reduce predictability of a random sequence, details of any planned restriction (eg, blocking) should be provided in a separate document that is unavailable to those who enrol participants or assign interventions	9
Allocation concealment mechanism	16b	Mechanism of implementing the allocation sequence (eg, central telephone; sequentially numbered, opaque, sealed envelopes), describing any steps to conceal the sequence until interventions are assigned	9
Implementation	16c	Who will generate the allocation sequence, who will enrol participants, and who will assign participants to interventions	9
Blinding (masking)	17a	Who will be blinded after assignment to interventions (eg, trial participants, care providers, outcome assessors, data analysts), and how	9
	17b	If blinded, circumstances under which unblinding is permissible, and procedure for revealing a participant's allocated intervention during the trial	N/A
**Methods: Data collection, management, and analysis**			
Data collection methods	18a	Plans for assessment and collection of outcome, baseline, and other trial data, including any related processes to promote data quality (eg, duplicate measurements, training of assessors) and a description of study instruments (eg, questionnaires, laboratory tests) along with their reliability and validity, if known. Reference to where data collection forms can be found, if not in the protocol	9-10

For peer review only - http://bmjopen.bmj.com/site/about/guidelines.xhtml

	18b	Plans to promote participant retention and complete follow-up, including list of any outcome data to be collected for participants who discontinue or deviate from intervention protocols	8
Data management	19	Plans for data entry, coding, security, and storage, including any related processes to promote data quality (eg, double data entry; range checks for data values). Reference to where details of data management procedures can be found, if not in the protocol	13
Statistical methods	20a	Statistical methods for analysing primary and secondary outcomes. Reference to where other details of the statistical analysis plan can be found, if not in the protocol	11-12
	20b	Methods for any additional analyses (eg, subgroup and adjusted analyses)	12
	20c	Definition of analysis population relating to protocol non-adherence (eg, as randomised analysis), and any statistical methods to handle missing data (eg, multiple imputation)	12
**Methods: Monitoring**			
Data monitoring	21a	Composition of data monitoring committee (DMC); summary of its role and reporting structure; statement of whether it is independent from the sponsor and competing interests; and reference to where further details about its charter can be found, if not in the protocol. Alternatively, an explanation of why a DMC is not needed	12
	21b	Description of any interim analyses and stopping guidelines, including who will have access to these interim results and make the final decision to terminate the trial	N/A
Harms	22	Plans for collecting, assessing, reporting, and managing solicited and spontaneously reported adverse events and other unintended effects of trial interventions or trial conduct	10
Auditing	23	Frequency and procedures for auditing trial conduct, if any, and whether the process will be independent from investigators and the sponsor	N/A
**Ethics and dissemination**			
Research ethics approval	24	Plans for seeking research ethics committee/institutional review board (REC/IRB) approval	4

For peer review only - http://bmjopen.bmj.com/site/about/guidelines.xhtml

Protocol amendments	25	Plans for communicating important protocol modifications (eg, changes to eligibility criteria, outcomes, analyses) to relevant parties (eg, investigators, REC/IRBs, trial participants, trial registries, journals, regulators)	14
Consent or assent	26a	Who will obtain informed consent or assent from potential trial participants or authorised surrogates, and how (see Item 32)	5
	26b	Additional consent provisions for collection and use of participant data and biological specimens in ancillary studies, if applicable	N/A
Confidentiality	27	How personal information about potential and enrolled participants will be collected, shared, and maintained in order to protect confidentiality before, during, and after the trial	12
Declaration of interests	28	Financial and other competing interests for principal investigators for the overall trial and each study site	13
Access to data	29	Statement of who will have access to the final trial dataset, and disclosure of contractual agreements that limit such access for investigators	12
Ancillary and post-trial care	30	Provisions, if any, for ancillary and post-trial care, and for compensation to those who suffer harm from trial participation	9-10
Dissemination policy	31a	Plans for investigators and sponsor to communicate trial results to participants, healthcare professionals, the public, and other relevant groups (eg, via publication, reporting in results databases, or other data sharing arrangements), including any publication restrictions	13
	31b	Authorship eligibility guidelines and any intended use of professional writers	13
	31c	Plans, if any, for granting public access to the full protocol, participant-level dataset, and statistical code	N/A

Appendices

For peer review only

Informed consent materials	32	Model consent form and other related documentation given to participants and authorised surrogates	Available in Swedish upon request. Could be translated upon reasonable request.
Biological specimens	33	Plans for collection, laboratory evaluation, and storage of biological specimens for genetic or molecular analysis in the current trial and for future use in ancillary studies, if applicable	N/A

*It is strongly recommended that this checklist be read in conjunction with the SPIRIT 2013 Explanation & Elaboration for important clarification on the items. Amendments to the protocol should be tracked and dated. The SPIRIT checklist is copyrighted by the SPIRIT Group under the Creative Commons "Attribution-NonCommercial-NoDerivs 3.0 Unported" license.

For peer review only

BMJ Open

Brief Self-guided Digital Intervention versus a Comprehensive Therapist-guided Online Cognitive Behavioral Therapy for Atopic Dermatitis: A Trial protocol for a Randomized Non-Inferiority Trial

Journal:	*BMJ Open*
Manuscript ID	bmjopen-2022-068908.R1
Article Type:	Protocol
Date Submitted by the Author:	01-Feb-2023

Complete List of Authors:	Kern, Dorian; Karolinska Institutet, Department of Clinical Neuroscience; Stockholm Health Care Services, Department of Clinical Neuroscience<br>Ljótsson, Brjánn; Karolinska Institutet, Department of Clinical Neuroscience, Division of Psychology<br>Lönndahl, Louise; Karolinska Institutet, Department of Medicine Solna<br>Hedman-Lagerlöf, Erik ; Karolinska Institute<br>Bradley, M; Karolinska Institutet<br>Lindefors, Nils; Karolinska Institutet, Department of Clinical Neuroscience<br>Kraepelien, Martin; Karolinska Institutet

**Primary Subject Heading**:	Dermatology
Secondary Subject Heading:	Health economics, Mental health
Keywords:	Eczema < DERMATOLOGY, Health economics < HEALTH SERVICES ADMINISTRATION & MANAGEMENT, Adult dermatology < DERMATOLOGY

SCHOLARONE™
Manuscripts

Trial protocol

Brief Self-guided Digital Intervention versus a Comprehensive Therapist-guided Online Cognitive Behavioral Therapy for Atopic Dermatitis: A Trial protocol for a Randomized Non-Inferiority Trial

For peer review only

Dorian Kern, MSc[1,2]., Brjánn Ljótsson, PhD[2], Louise Lönndahl[3], MD, PhD., Erik Hedman-Lagerlöf[2], PhD[1], Maria Bradley, MD, PhD[3], Nils Lindefors MD, PhD[1], Martin Kraepelien, PhD[1,2]

[1]Centre for Psychiatry Research, Department of Clinical Neuroscience, Karolinska Institutet & Stockholm Health Care Services, Region Stockholm, Norra Stationsgatan 69, SE-113 64 Stockholm, Sweden

[2]Division of Psychology, Department of Clinical Neuroscience, Karolinska Institutet, Nobels väg 9 SE-171 65 Solna, Sweden

[3]Dermatology and Venereology Unit, Department of Medicine Solna, Karolinska Institutet, SE-171 77 Stockholm, Sweden

Correspondence: Dorian Kern; Centre for Psychiatry Research, Norra stationsgatan 69, 113 64, Stockholm; +467 23 24 32 35, dorian.kern@ki.se

Key words: Atopic dermatitis; eczema; pruritus; cognitive behavioral therapy; self-care

Word count: 4173

Version: 1.2

Date: 2022-28-09

Abstract

Introduction Our aim is to investigate whether a shortened digital self-care intervention is noninferior to, and cost-effective compared with, a comprehensive and therapist-guided CBT treatment for AD.

Methods and analysis This is a single-blind, randomized clinical non-inferiority trial at Karolinska Institutet, a medical university in Stockholm, Sweden. We will recruit 174 adult participants with AD through self-referral. Participants will be randomized 1:1 to the two experimental conditions. Participants randomized to guided care will receive internet-delivered cognitive behavior therapy for 12 weeks. Participants randomized to digital self-care will have access to this self-guided intervention for 12 weeks. At post-treatment (primary endpoint), non-inferiority will be tested and resource use will be compared between the two treatment groups. Cost-effectiveness will be explored at 1-year follow-up. Potential mediators will be investigated. Data will be analyzed intention-to-treat. We define non-inferiority as a 3-point difference on the primary outcome measure (Patient-oriented Eczema Measure). Recruitment is planned to start in late autumn 2022.

Ethics and dissemination This study is approved by the Swedish ethics authority (reg. no 2021-06704-01) and is preregistered at ClinicalTrials.gov. The study will be reported according to the Consolidated Standards of Reporting Trials statement for non-pharmacological trials. The results of the study will be published in peer-reviewed scientific journals and disseminated to patient organizations and media.

Trial registration: ClinicalTrials.gov identifier: NCT05517850, https://clinicaltrials.gov/ct2/show/NCT05517850

Strengths and limitations of this study

- First study comparing self-care and therapist guided cognitive behavioral therapy (CBT) for atopic dermatitis (AD).
- Broad inclusion criteria will help generalize the results to a variety of AD cases.
- Depending on what time of the year the majority of participants eventually will be recruited, it may be difficult to control for seasonal changes in Sweden's temperate climate.

For peer review only

- Earlier similar studies indicate a risk of bias for the recruitment of female participants with an advanced education.
- The self-guided condition is different from the therapist-guided in several ways, including no therapist and shorter content. If the self-guided condition cannot be found non-inferior, this study cannot differentiate what factors caused that result.

Background

Atopic dermatitis

Atopic dermatitis (AD) is the most prevalent of the inflammatory skin diseases. The primary symptoms of AD are itch, dry skin, and rash[1]. Treatment and prevention generally consist of emollient creams, topical corticosteroids, other local immunosuppressive creams such as calcineurin inhibitors. If topical treatment is ineffective, phototherapy and systemic immunomodulatory treatments are often given[2]. In addition to dermatological symptoms, people with AD have an increased risk of major depression and anxiety disorders[3, 4]. Furthermore, behavioral and emotional factors may affect AD symptoms. A common reaction to the itching sensation of the skin is the act of scratching, which, while temporarily relieving, can lead to damage to the skin barrier and an aggravated inflammation[5]. Furthermore, people with AD tend to scratch when they experience anxiety or depressed mood[6], possibly as a conditioned behavior. In addition, avoidance of itch-associated situations is frequently documented, which may lead to avoidance of activities that are otherwise appreciated or important to the patient, which can further affect mental health and quality of life[7] The impairment of quality of life by AD is comparable to asthma and type 1 diabetes[2]. The facts that AD can affect mental health, and that behavior can directly affect AD symptoms, make psychological treatment relevant.

Educational and psychological interventions for atopic dermatitis

Various forms of educational and psychological support may be available for people with AD. According to structured reviews, studies of educational interventions for AD have often had suboptimal methodology and unclear results, with tendencies towards effect on skin-related quality of life and improved symptoms[8]. These interventions

For peer review only

may be held in a group or individual setting and may be led by physicians, nurses or other professionals. One recent RCT of an Internet-delivered educational and behavioral intervention, including young adults, showed small but significant effects on AD symptoms and secondary measures [9].

Internet-delivered cognitive behavioral therapy

Internet cognitive-behavioral therapy (ICBT) is a psychological treatment administered over the Internet, otherwise similar to regular face-to-face CBT. ICBT usually includes therapist support via a secure digital communication, similar to e-mail. Studies have shown that a clinician spends the equivalent of about fifteen minutes per patient and week in ICBT[10]. ICBT has been suggested to improve cost-effectiveness as well as the availability of evidence-based treatment[11] and research reviews show that there are no consistent differences in effectiveness between conventional face-to-face CBT and ICBT[12]. In Sweden, ICBT has been offered in regular care via the Internet Psychiatry Unit in Stockholm since 2007[13].

There is an unmet need for interventions for people with mild to moderate illness, where a comprehensive and therapist-guided ICBT program would be an unnecessarily intense level of care, and thus an inefficient use of resources. One solution is to use existing ICBT programs and simply remove the therapist-guidance. Some research has suggested that this has negative impact on effectiveness, however, there are indications that the amount and type of therapist contact are not decisive[14] and recent studies have compared therapist supported ICBT and self-guided ICBT and shown that treatment outcome, treatment implementation and treatment satisfaction do not differ between conditions if certain components are present in the self-guided intervention. These components include a stringent clinical process in the form of an assessment and a follow-up interview with a clinician and a high-quality interface[15, 16].

Internet-delivered cognitive behavioral therapy for atopic dermatitis

An ICBT protocol has been evaluated for AD. The main components were symptom diary, mindfulness, and exposure to avoided sensations and situations, with prevention of unhelpful responses. The treatment showed promising preliminary effects in a face-

For peer review only

to-face pilot study, which subsequently was confirmed in a randomized controlled trial (RCT)[17, 18]. A digital self-care intervention has been preliminarily evaluated for people with AD, with promising effects[19]. Digital self-care was based on the ICBT intervention with comparable components. Thus, there is support that CBT can be effective in improving mental health and reducing symptoms of AD, although additional studies are needed. A comparison between the ICBT and reworked digital self-care variants could further contribute to the literature and the results will have implications for implementation.

This is a study protocol of a single-blind non-inferiority RCT, aimed at evaluating the effects of a self-guided CBT program available via the internet (digital self-care) for people with AD, in relation to a previously evaluated therapist-guided internet-delivered CBT program (guided care)[18]. The main hypothesis is that digital self-care will be non-inferior to guided care in reducing self-rated eczemic symptoms, as measured by the Patient-oriented eczema measure (POEM)[20]. Secondary purposes are to evaluate use of resources and cost-effectiveness of digital self-care compared to guided care, and to investigate potential mediators of symptom improvement.

Hypotheses

Primary hypothesis: Digital self-care will be non-inferior to ICBT, according to the predefined non-inferiority margin of 3 points, as measured by the primary outcome (POEM).

Secondary hypothesis 1: Digital self-care will be associated with significantly less staff costs than ICBT, in the form of time spent in hours by a licensed psychologist.

Secondary hypothesis 2: Digital self-care will be cost-effective compared to ICBT, assessed at one-year follow-up.

Secondary hypothesis 3: Effects on the outcome will be mediated by the amount of time spent by participants on main treatment components.

For peer review only

Study design

Timeframe

The first participant was included 2022-29-11. The first cohort started 2022-05-12. Post treatment data collection is expected to finish summer or autumn 2023, with the last follow-up a year after that. The present study is a non-inferiority RCT for adults with AD. Participants randomized to guided care will receive ICBT via the internet, guided by a therapist for 12 weeks. Participants randomized to digital self-care get access to a self-guided CBT-based intervention for 12 weeks. Condition allocation will be 50/50.

The primary end point is at 12 weeks, (i.e., post-treatment). The study will also include a six-month and a one-year follow-up. 1 year after treatment conclusion, long-term effects will be evaluated. An overview of the trial design can be seen in Figure 1.

The study is approved by the Swedish ethics authority (reg. no 2021-06704-01) and is be preregistered at ClinicalTrials.gov. The participants will receive written and verbal information about the study. The participants will digitally sign an informed consent in a secure platform.

INSERT FIGURE 1 HERE

Participants

Participants will be recruited via advertisements in social media and with the help of relevant patient associations' mailing lists and websites. Interested individuals with self-reported AD diagnosis may participate following their written consent to the study. Advertisements in different types of social media has the advantage that it reaches different types of people regarding age and gender. In some platforms, it is possible to make the advertisements to only appear for men, if the female percentage should be considered too high, which is something we could consider. In order to minimize false diagnoses, participants will disclose at which point in time they received their AD diagnosis, and if they can recall, at what clinic and by which physician. They will then receive login information to access a secure digital platform, where data from self-assessment scales will be registered. Participation in the study does not involve any change in their regular treatment or medical self-care. Participants will be evaluated by

For peer review only

a licensed psychologist in a clinical interview, assessing suitability according to the inclusion criteria and motivation. At the end of the interview, the interviewer explains that participation will require considerable effort, and asks if the potential participant believes this to be reasonable. If the potential participant answers yes, the interviewer formally asks if they are interested study participation. If a potential participant states their interest, acknowledging that these interventions will require considerable time and effort, that participant is considered motivated.

Inclusion criteria: At least 18 years of age with self-reported AD diagnosis. No new types of medications introduced for six months prior, with no intention of future change (participants will not be excluded if they change their medication after inclusion).Participants need to be able to understand written and spoken Swedish, have access to the internet and be able to receive telephone calls and text messages.

Exclusion criteria: Other disease or condition that has immediate treatment priority over AD. This could be an ongoing demanding treatment of a severe somatic or psychiatric condition. No specific diagnoses are inevitably a cause for exclusion, as potential participants will rather be evaluated on a case-to-case basis.

Setting

The trial is conducted at Karolinska Institutet, a medical university in Stockholm, Sweden. Dermatologists involved in the study are also associated with the dermatology clinic at Karolinska University Hospital, in Stockholm, Sweden. Initial assessment will be made by telephone by a clinical psychologist, and both study arms are entirely conducted online, including the collection of self-rated assessments. Participants will be recruited from all parts of Sweden.

Power calculation

Based on data from Hedman-Lagerlöf and colleagues[17] and Kern and colleagues[18] we expect an in-group effect of about d=0.85 for both interventions. A consultant statistician performed a power calculation. The required sample size was computed based on boot-strapped (5000 simulations) reruns of weekly measured data obtained in the therapist-guided RCT[17]. The results indicated that a sample size of 174 would be

For peer review only

required for 90% power, α=.05, given a non-inferiority margin of 3 points on the POEM scale and accounting for 10% missing data.

Planned interventions

Usual care

Participants will continue their usual care. According to national guidelines, people with AD should be treated according to a stepped-care model, with emollients and education at the bottom, advancing to cortisone treatments, up to more advanced treatments, such as immunosuppressive and biological interventions. Between individuals, usual care in Sweden can vary greatly. People with AD in Sweden may be treated and followed up at primary or specialist levels. Not all people with AD have regular contact with a physician at all, some have intermittent check-ups, and others may have close and frequent contact. Psychological interventions are not usually available, whereas educational interventions may be available in some regions of Sweden.

Guided care

The aim of CBT for AD is to target behavioral and emotional processes, to improve AD symptoms and increase quality of life. The participants read education material and uses worksheets and homework reports. The material is in Swedish and is currently only available to study personnel and participants. This intervention consists of 10 modules and lasts 12 weeks. An introduction provides information about CBT and AD. Participant register itching and scratching behaviors, with the aim to increase awareness of these behaviors. Gradually, the participants are introduced to the proposed active treatment components: mindfulness training, and exposure with response prevention. Mindfulness is trained throughout the intervention. The purpose of mindfulness is to increase awareness of emotional and behavioral reaction patterns, and to increase tolerance of aversive inner experiences[21]. Additionally, mindfulness training may improve stress tolerance. Participants are also trained in self-compassion, to increase understanding of one's own reactions and to increase the ability to handle difficult situations in the treatment and life[22]. After practicing these fundamental skills, exposure with response prevention is introduced in week 4. Exposure is a

For peer review only

controlled way to gradually approach situations that evoke strong emotions or somatic reactions. The purpose is to gain knowledge and experience in how this approach can change one's perception of unwanted situations, emotions, or somatic reactions[23]. The assumption is that skills learned from mindfulness practice and self-compassion training helps the patient to conduct exposure exercises. Response prevention means to reduce scratching in these problematic situations in order to reduce itch in the long term. Through this whole process, the participant is guided by a therapist. The therapist may be contacted through a secure internet platform at any time and responds within 48 hours. Participants read the education material, construct exercises to perform, describe their progress in homework assignments, and receive feedback from their therapist. The therapists are all licensed psychologists, with experience of treating people with somatic conditions, and experience of treating via the Internet in a text-based format. During the 12-week period, the participants work at their own pace, and contacts their therapist when necessary. The recommended pace is however one module per week, and the therapist contact participants who are inactive. All participants have access to the same treatment material but will construct exposure exercises that fit their specific situation, assisted by the therapist. The therapists will guide participants to make exercises as faithful to the treatment as possible. Participants will report back what they have done. Because therapist and participants never meet, there is no control of whether participants actually perform exercises as they are reported. The study coordinator will offer guidance to other therapists if needed.

Digital self-care

As with guided care, the aim of CBT for AD is to target behavioral and emotional processes, to improve AD symptoms and increase quality of life. The material is similar to guided care but shortened. Likewise, participants, read information, and perform exercises in their everyday life. As with guided care, the main treatment components are mindfulness and exposure with response prevention, with a specific focus on itch. Participants will be able to practice mindfulness with the help of instructions and examples. Because it may be challenging to construct relevant exposure exercises without the help of a therapist, participants will receive detailed instructions and guidance, and get to choose exposure exercises from a long list of examples. They will also be free to create their own relevant exposure situations within the intervention.

For peer review only

Digital self-care consists of 8 modules. The only active interventions, mindfulness and exposure are introduced week 1 and 2, respectively. From week 3-7, no new interventions are introduced and participants are advised to continue practicing the interventions, but there is a new theme of education each week, to act as inspiration for exercises. The last 4 weeks, the participant is merely advised to continue practice. There is no therapist, but the study coordinator monitors the process and may contact inactive participants. Participants will access the same digital platform as the guided condition. Participants work entirely at their own pace. Modules will be unlocked automatically if one week has passed, and all material in the previous module has been read. Participants will report if they have done their assignments, but there is no control if they do as they report.

Differences between study conditions

The main differences between the conditions are that digital self-care a) has contact with a clinician at only two points in time: pre intervention and after eight weeks plus the addition of automated messages, whereas guided care has continuous contact throughout the treatment via text messages on a secure platform, and that b) the treatment text is significantly shortened. Automated emails are sent every week to the non-guided group, consisting of a greeting, and some encouraging words relevant to the week's content. Both groups receive automated text messages at the time of new assessments. Guided care will also have an evaluation from a clinician via telephone at two points in time. Furthermore, digital self-care uses a mobile optimized interface, and have improvements in design aimed to decrease the need of guidance.

Therapists

Only the guided group will have therapist contact. The self-guided group will be managed by the study coordinator. The therapists are licensed psychologists specialized in CBT. All therapists will participate in an introductory workshop before treating in the study. The project coordinator will supervise the therapists regularly and on-demand.

Randomization/blinding

Participants will be blinded to condition. They will be generally informed that they will be randomized to “either one of two types of self-help interventions based on CBT”, not

knowing any difference between the conditions. After baseline measurements, included participants will be allocated randomly (1:1) to therapist-guided or self-guided treatment in consecutive even-numbered blocks. Randomization will be based on a true random number service and conducted by a person blinded to the participants, who is not otherwise involved with the study, using a true random number generator (http://www. random.org). It is impossible for the therapists performing the inclusion assessment to anticipate participant allocation.

Measurements

The outcome measures are based on those used previously by Hedman-Lagerlöf and colleagues[17] and Kern and colleagues[18], for the evaluation of CBT interventions for AD. The battery of instruments in the current study overlaps with the recently published HOME criteria (harmonizing measures for eczema), but have several additional measures, and does not include some of the suggested measures [24]. We do include the suggested measurements for self-reported symptoms and quality of life but have no clinician-rated measurement of symptoms, which we acknowledge as a weakness.

Primary outcome measure: Patient Oriented Eczema Measure (POEM)[20] measures the severity of symptoms of atopic dermatitis, which has a range of 0 to 28. The POEM has excellent psychometric properties and is widely used[25]. In this study, in order to be conservative, response is operationalized as a POEM score reduction 4 points or more, and the non-inferiority margin is operationalized as 3 points. This is the same as earlier studies of CBT for AD, based on changes from severe eczema. Another evaluation has found smaller changes, around 2.1 to 2.9 as "likely to be beyond measurement error but unlikely to be clinically important"[26]. Because of this, as we are including participants with mild, moderate and severe eczema, we could possibly have chosen an even smaller margin.

Secondary outcome measures: Demographical data will be collected upon sceening. Peak pruritus numerical rating scale (NRS)[27], is used to measuree itching sensations; Perceived Stress Scale (PSS)[28] is used to measure general experience of stress; Patient Health Questionnaire-9 (PHQ-9)[29] is used to measure depressive symptoms; Dermatological Life Quality Index (DLQI)[30] is used to measure AD-specific quality of life; Insomnia severity index (ISI)[31] is used to measure severity of sleep problems and

For peer review only

possible insomnia; Client Satisfaction Questionnaire (CSQ-8)[32] is used to measure how satisfied participants are with the intervention. We will also ask participants to report if they experience adverse events associated with the treatment (AE). In addition to this, we will use the Credibility / Expectancy Scale (CES)[33] to measure credibility. A the end of the intervention, the participants will be asked to estimate the time spent in the intervention (ToE). Resource use measured by the time the clinician spends on the different tasks associated with administering the intervention, doing interviews, and giving support. Euro-QOL 5 dimensions (EQ-5D)[34] will be used to assess quality of life from a health economics perspective. Treatment Inventory of Costs in Psychiatric Patients (TIC-P)[35] will be used to assess health-care costs.

Outcomes at all scales will be compared with available published results regarding other Internet-based interventions for each scale.

Measurement points

Please refer to table 1.There are no planned interim analyses. There are no predefined criteria for trial termination.

INSERT TABLE 1 here

Safety considerations

Participants' regular healthcare is unaffected by either of the study conditions. Possible benefits for patients with increased ability to manage their disease are presumed to justify any disadvantages in the form of time consumption and temporary discomfort. Adverse treatment effects will be examined both during the intervention and after the total twelve weeks of the intervention. If the disease worsens, the participants will be encouraged to seek help from their regular specialist clinic or primary health care center. All communication and data storage take place on an encrypted and secure digital platform and is in accordance with the Karolinska Institutet's regulations and the EU General Data Protection Regulation. In case of any serious harm, the standard patient's insurance for Government financed health care will be applicable to every participant.

For peer review only

Statistical analysis

Non-inferiority margin

Earlier research[22] has defined clinically significant improvement on the main outcome measure to be 3,4 points, which is the basis for our non-inferiority margin of 3 points.

Non-inferiority

Change over time will be modelled using mixed-effects linear regression with a random intercept and slope (time). The main outcome POEM will be analyzed by the regression on time (baseline to end point), group (digital self-care vs guided care), and the interaction of time and group. We will consider digital care to be non-inferior to therapist-guided if the upper bound of the 1-sided 95% confidence interval for the time × group interaction ($\alpha = .05$) is within the a-priori non-inferiority margin of 3 points on the POEM[36]. In addition, we will investigate non-inferiority in a sensitivity analysis per-protocol framework with data from patients who will have initiated module 4 or further in either of the study arms. As for interpretation of results, if the two-sided 95% confidence interval does not contain zero, this will be viewed as indicative of superiority of the corresponding treatment. We will estimate Cohen's *d* by dividing the time × group coefficient by the pooled observed baseline standard deviation. All primary analyses will be conducted intent-to-treat . The power calculations allow for 10% missing post-treatment data on the primary outcome at 90% power. If we have more than 10% missing data, we will use multiple imputation before conducting the primary analysis."

Resource utilization

At the primary endpoint, resource utilization will be preliminary assessed by measuring therapist time for each treatment condition and compared with a typical salary for a clinical psychologist employed in Stockholm, Sweden.

For peer review only

Cost-effectiveness

At one-year follow-up, a cost-effectiveness analysis will be performed from a-healthcare system perspective, based on recorded therapist time, as well as from a societal perspective. The incremental cost-effectiveness ratio will be estimated at post-treatment and at follow-up. We will also use the TIC-P to collect economic cost data[34], together with the EQ-5D[35]. The costs will be assessed in Swedish krona and converted into US dollar using 2023 as the reference year. The costs of the self-guided and therapist-guided treatments will be modelled as a function of therapist time using the same healthcare index to determine tariffs for licensed psychologists. According to our hypothesis, there should be a significant difference in treatment costs but there should not be any significant difference in societal costs. We will calculate quality-adjusted life-years using the area under the curve approach[36], use regression analyses to analyze costs[37], and use nonparametric bootstrapping with 5000 iterations, where differences in costs and outcomes will be paired. The incremental cost-effectiveness ratio will be used as a cost-effectiveness estimate[38].

Mediation

We are interested in the mediating effect of time spent on mindfulness and exposure exercises on the outcome. Because we have a non-inferiority hypothesis, we will not perform traditional mediation analysis where the mediator is proposed to account for between-group differences. Instead, we will do within-group process analysis to investigate if treatment activity predicts simultaneous and/or subsequent change in POEM during treatment[39]. The exact analytic model will be determined based on the nature of data that we are able to collect on treatment activity. We will also explore potential moderated mediation, i.e., if mediators differ between the two types of treatment. We also intend to analyze self-rated pruritus, perceived stress, and depressed mood as potential mediators.

Data

All data will be stored at a secure digital platform, to which only study personnel have access. Measurements from the participants will be submitted in the platform itself. Because all data is submitted and stored in the same digital platform, we will not use a

For peer review only

data monitoring committee. The final trial data set will be primarily handled by the first, second and final author. However, all authors will be granted access to the data set if necessary. We have no plans to make an individual-level data set or any statistical code public. Data will be available upon reasonable request to the corresponding author.

Patient and public involvement

We have used input from participants in the feasibility trial[19], on which we based intervention improvements in the present study. This included various revisions, the most important of which was to add a "scratch-free time" intervention. The duration is defined by the participant, and gradually increased. This ensured an easier start compared to the previous variant to begin with an entire scratch-free day. Some participants also wanted therapist guidance, which could not be considered for this particular intervention. We also considered feedback from a patient representative, who was not a trial participant. They largely agreed with the feedback from trial participants, encouraged the intervention in its current form, and furthermore participated in an introductory seminar with the treating study psychologists. In the current trial, no participants were involved in decisions about study design, outcome measures, or research question. A patient organization for AD, as well as one for disorders related to asthma and allergies (Atopikerna and Astma & Allergiförbundet) will be involved in the recruitment of participants by informing their members about the study. We plan to disseminate the results of the research to study participants and to the aforementioned patient organizations.

Ethics and Dissemination

The study will be carried out according to the predefined standards of this trial protocol, the Declaration of Helsinki and Good Clinical Practice. This study will be reported according to the CONSORT statement for non-pharmacological trials[40]. The interventions that will be administered have deemed safe by consultant dermatologists and have been previously evaluated without serious adverse events[18,19]. This study will use the Vancouver criteria for authorship. The results of this study will be published in peer-reviewed scientific journals, be presented to the scientific community

For peer review only

at conferences. The results will also be communicated to study participants, patient organizations and clinicians.

Acknowledgement

Author contributions

Dorian Kern: Study design, intervention design, drafting of manuscript, planning of statistical analysis. Brjánn Ljótsson: Study design, intervention design, planning of statistical analysis. Louise Lönndahl: Contributions to intervention and manuscript; Maria Bradley: Study concept and contributions to manuscript. Erik Hedman-Lagerlöf: Study concept, contributions to manuscript and planning of statistical analysis; Nils Lindefors: Study concept, study design, contributions to manuscript. Martin Kraepelien: Study concept, contributions to manuscript, intervention design, study design, planning of statistical analysis, principal investigator. All authors: Critical review and revision of manuscript.

Funding/support

This study will be funded in part by the Swedish Ministry of Health and Social affairs. The funder has no role in the design and conduct of the study and will have no influence of data collection, management, analysis, interpretation. The funder will have no part in preparation, review or approval of the manuscript, or the decision to submit the manuscript for publication.

Declaration of conflict of interest

Dr Ljótsson reported being a shareholder of DahliaQomit AB and holding copyright for a cognitive behavioral treatment manual for irritable bowel syndrome, with royalties paid from Pear Therapeutic, Inc, outside of submitted work. Dr Lönndahl reported receiving a personal fee from LEO Pharma and Sanofi Genzyme outside of submitted work. Dr Hedman-Lagerlöf reported being a shareholder of DahliaQomit AB and holding copyright for a cognitive behavioral treatment manual for irritable bowel syndrome, with royalties paid from Pear Therapeutic, Inc, outside of submitted work. No other disclosures were reported.

For peer review only

References

1. Drucker AM, Wang AR, Li WQ, et al. The Burden of Atopic Dermatitis: Summary of a Report for the National Eczema Association. *J Invest Dermatol*. 2017;137(1):26-30. doi:10.1016/j.jid.2016.07.012
2. Weidinger S & Novak N. Atopic dermatitis. *Lancet*. 2016;387(10023):1109-1122. doi:10.1016/S0140-6736(15)00149-X
3. Yaghmaie P, Koudelka CW, Simpson EL. Mental health comorbidity in patients with atopic dermatitis. *J Allergy Clin Immunol.* 2013; 131(2): 428-33
4. Sandhu JK et al, Wu KK, Bui T-L, Armstrong AW. Association Between Atopic Dermatitis and Suicidality: A Systematic Review and Meta-analysis. *JAMA Dermatology*. 2019; 155(2): 178-87.
5. Wahlgren CF. Itch and atopic dermatitis: an overview. *J Dermatol.* 1999; 26(11): 770-9
6. Daunton A, Bridgett C, Goulding JM. Habit reversal for refractory atopic dermatitis: a review. *Br J Dermatol.* 2016; 174(3): 657-9.
7. Verhoeven L, Kraaimaat F, Duller P, et al. Cognitive, behavioral, and physiological reactivity to chronic itching: analogies to chronic pain. *International journal of behavioral medicine.* 2006; 13(3): 237-43.
8. Pickett K, Loveman E, Kalita N, Frampton G, Jones J. Educational interventions to improve quality of life in people with chronic inflammatory skin diseases: systematic reviews of clinical effectiveness and cost-effectiveness. *Health Technology Assessment*. 2015;19(86):i-176.
9. Santer M, Muller I, Becque T, Stuart B, Hooper J, Steele M et al. Eczema Care Online behavioural interventions to support self-care for children and young people: two independent, pragmatic, randomised controlled trials *BMJ* 2022; 379 :e072007 doi:10.1136/bmj-2022-072007
10. Hedman E, Ljótsson B, Kaldo V, et al. Effectiveness of Internet-based cognitive behaviour therapy for depression in routine psychiatric care. *J Affect Disord*. 2014;155:49-58. doi:10.1016/j.jad.2013.10.023

11. Cavanagh K. Geographic inequity in the availability of cognitive behavioural therapy in England and Wales: a 10-year update. *Behav Cogn Psychother*. 2014;42(4):497-501. doi:10.1017/S1352465813000568

12. Carlbring P, Andersson G, Cuijpers P, et al. Internet-based vs. face-to-face cognitive behavior therapy for psychiatric and somatic disorders: an updated systematic review and meta-analysis. *Cogn Behav Ther*. 2018;47(1):1-18. doi:10.1080/16506073.2017.1401115

13. Lindefors N, El Alaoui S, Svanborg C. Internet-KBT ger effektiv behandling - Signifikant hållbar förbättring vid depression, paniksyndrom och socialt ångestsyndrom [Internet CBT provides effective treatment]. *Läkartidningen*. 2017;114:D77R. Published 2017 Jan 24.

14. Hirai M, Clum GA. A meta-analytic study of self-help interventions for anxiety problems. *Behav Ther*. 2006;37(2):99-111. doi:10.1016/j.beth.2005.05.002

15. Dear BF, Staples LG, Terides MD, et al. Transdiagnostic versus disorder-specific and clinician-guided versus self-guided internet-delivered treatment for Social Anxiety Disorder and comorbid disorders: A randomized controlled trial. *J Anxiety Disord*. 2016;42:30-44. doi:10.1016/j.janxdis.2016.05.004

16. Titov N, Dear BF, Staples LG, et al. Disorder-specific versus transdiagnostic and clinician-guided versus self-guided treatment for major depressive disorder and comorbid anxiety disorders: A randomized controlled trial. *J Anxiety Disord*. 2015;35:88-102. doi:10.1016/j.janxdis.2015.08.002

17. Hedman-Lagerlöf E, Bergman A, Lindefors N, et al.. Exposure-based cognitive behavior therapy for atopic dermatitis: an open trial. *Cogn Behav Ther*. 2019;48(4):300-310. doi:10.1080/16506073.2018.1504320

18. Hedman-Lagerlöf E, Fust J, Axelsson E, et al. Internet-Delivered Cognitive Behavior Therapy for Atopic Dermatitis: A Randomized Clinical Trial. *JAMA Dermatol*. 2021;157(7):796-804. doi:10.1001/jamadermatol.2021.1450

19. Kern D, Ljótsson B, Lönndahl L, et al. Optimized User Experience, Efficiency, and Resource Use in Online Self-Management of Atopic Dermatitis. *JAMA Dermatol.* 2022;158(11):1325–1327. doi:10.1001/jamadermatol.2022.3434

20. Charman CR, Venn AJ, Williams HC. The Patient-Oriented Eczema Measure: development and initial validation of a new tool for measuring atopic eczema severity from the patients' perspective. *Arch Dermatol* 2004; 140:1513–19.

21. Hayes SC, Luoma JB, Bond FW, et al,. Acceptance and commitment therapy: model, processes and outcomes. *Behav Res Ther*. 2006;44(1):1-25. doi:10.1016/j.brat.2005.06.006
22. Clarke EN, Thompson AR, Norman P. Depression in people with skin conditions: The effects of disgust and self-compassion. *Br J Health Psychol*. 2020;25(3):540-557. doi:10.1111/bjhp.12421
23. Sewart AR, Craske MG. Inhibitory learning. In: Abramowitz JS, Blakey SM, editors. Clinical handbook of fear and anxiety: Maintenance processes and treatment mechanisms. Washington, DC, USA: American Psychological Association; 2020:265-285.
24. Williams HC, Schmitt J, Thomas KS, et al. The HOME Core outcome set for clinical trials of atopic dermatitis. *J Allergy Clin Immunol*. 2022;149(6):1899-1911. doi:10.1016/j.jaci.2022.03.017
25. Schram ME, Spuls PI, Leeflang MM, et al. EASI, (objective) SCORAD and POEM for atopic eczema: responsiveness and minimal clinically important difference. *Allergy*. 2012;67(1):99-106. doi:10.1111/j.1398-9995.2011.02719.x
26. Howells L, Ratib S, Chalmers JR, Bradshaw L, Thomas KS, CLOTHES trial team. How should minimally important change scores for the Patient-Oriented Eczema Measure be interpreted? A validation using varied methods. *British Journal of Dermatology.* 2018 May;178(5):1135-42.
27. Yosipovitch G, Reaney M, Mastey V, et al. Peak Pruritus Numerical Rating Scale: psychometric validation and responder definition for assessing itch in moderate-to-severe atopic dermatitis. *Br J Dermatol*. 2019;181(4):761-769. doi:10.1111/bjd.17744
28. Cohen S, Kamarck T, Mermelstein R. A global measure of perceived stress. *J Health Soc Behav*. 1983;24(4):385-396.
29. Kroenke K, Spitzer RL, Williams JB. The PHQ-9: validity of a brief depression severity measure. *J Gen Intern Med.* 2001; 16(9): 606-13.
30. Finlay AY, Khan GK. Dermatology Life Quality Index (DLQI)--a simple practical measure for routine clinical use. *Clin Exp Dermatol.* 1994; 19(3): 210-6.
31. Morin, C.M. Insomnia: Psychological Assessment and Management. Guilford, New York; 1993

For peer review only

32. Attkisson CC, Zwick R. The client satisfaction questionnaire. Psychometric properties and correlations with service utilization and psychotherapy outcome. *Eval Program Plann*. 1982;5(3):233-237. doi:10.1016/0149-7189(82)90074-x

33. Devilly GJ, Borkovec TD. Psychometric properties of the credibility/expectancy questionnaire. *J Behav Ther Exp Psychiatry*. 2000;31(2):73-86. doi:10.1016/s0005-7916(00)00012-4

34. Bouwmans C, De Jong K, Timman R, et al. . Feasibility, reliability and validity of a questionnaire on healthcare consumption and productivity loss in patients with a psychiatric disorder (TiC-P). BMC Health Serv Res. 2013;13(1):217. doi:10.1186/1472-6963-13-217 - DOI - PMC - PubMed

35. Rabin R, de Charro F. EQ-5D: a measure of health status from the EuroQol Group. *Ann Med*. 2001;33(5):337-343. doi:10.3109/07853890109002087

36. Walker J. Non-inferiority statistics and equivalence studies. *BJA Educ*. 2019;19(8):267-271. doi:10.1016/j.bjae.2019.03.004

37. Matthews JNS, Altman DG, Campbell MJ, et al. Analysis of serial measurements in medical research. BMJ. 1990;300(6719):230-235. doi:10.1136/bmj.300.6719.230 - DOI - PMC - PubMed

38. Manning WG, Mullahy J. Estimating log models: to transform or not to transform?. *J Health Econ*. 2001;20(4):461-494. doi:10.1016/s0167-6296(01)00086-8

39. H. Hesser. Modeling individual differences in randomized experiments using growth models: recommendations for design, statistical analysis and reporting of results of internet interventions Internet Interv. 2015, 110-120, 10.1016/j.invent.2015.02.003

40. Schulz KF , Altman DG , Moher D . CONSORT 2010 Statement: updated guidelines for reporting parallel group randomised trials. *BMC Med* 2010;8:18.doi:10.1186/1741-7015-8-18

Table 1. Measurement points

Screening	Pre	Weekly	Week2	MID	POST	FU6	FU12
Demo	POEM	POEM	CES	POEM	POEM	POEM	POEM
POEM	NRS	NRS		NRS	NRS	NRS	NRS
NRS	DLQI	DLQI		DLQI	DLQI	DLQI	DLQI
DLQI	PHQ-9	PHQ-9		PHQ-9	PHQ-9	PHQ-9	PHQ-9
PHQ-9	PSS	PSS		PSS	PSS	PSS	PSS
	ISI	ISI		TIC-P	TIC-P	TIC-P	TIC-P
	TIC-P			EQ-5D	EQ-5D	EQ-5D	EQ-5D
	EQ-5D			ISI	ISI	ISI	ISI
					CSQ-8		
					AE		
					ToE		

Table 1 Abbreviations: Demo = demographical data; POEM = Patient Oriented Eczema Measure; NRS = Peak Pruritus Numerical Rating Scale; DLQI = Dermatological Life Quality Index; PHQ-9 = Patient Health Questionnaire 9 item; PSS = Perceived Stress Scale; ISI = Insomnia Severity Index; TIC-P = Treatment Inventory of Costs in Psychiatric Patients; EQ-5D = Euro-QOL 5 dimensions; CES = Credibility/Expectancy Scale; AE = Adverse Events; ToE = Time (spent) on Exercises

Figure 1 legend: Consort 2010 flow diagram

Figure 1 CONSORT 2010 Flow Diagram

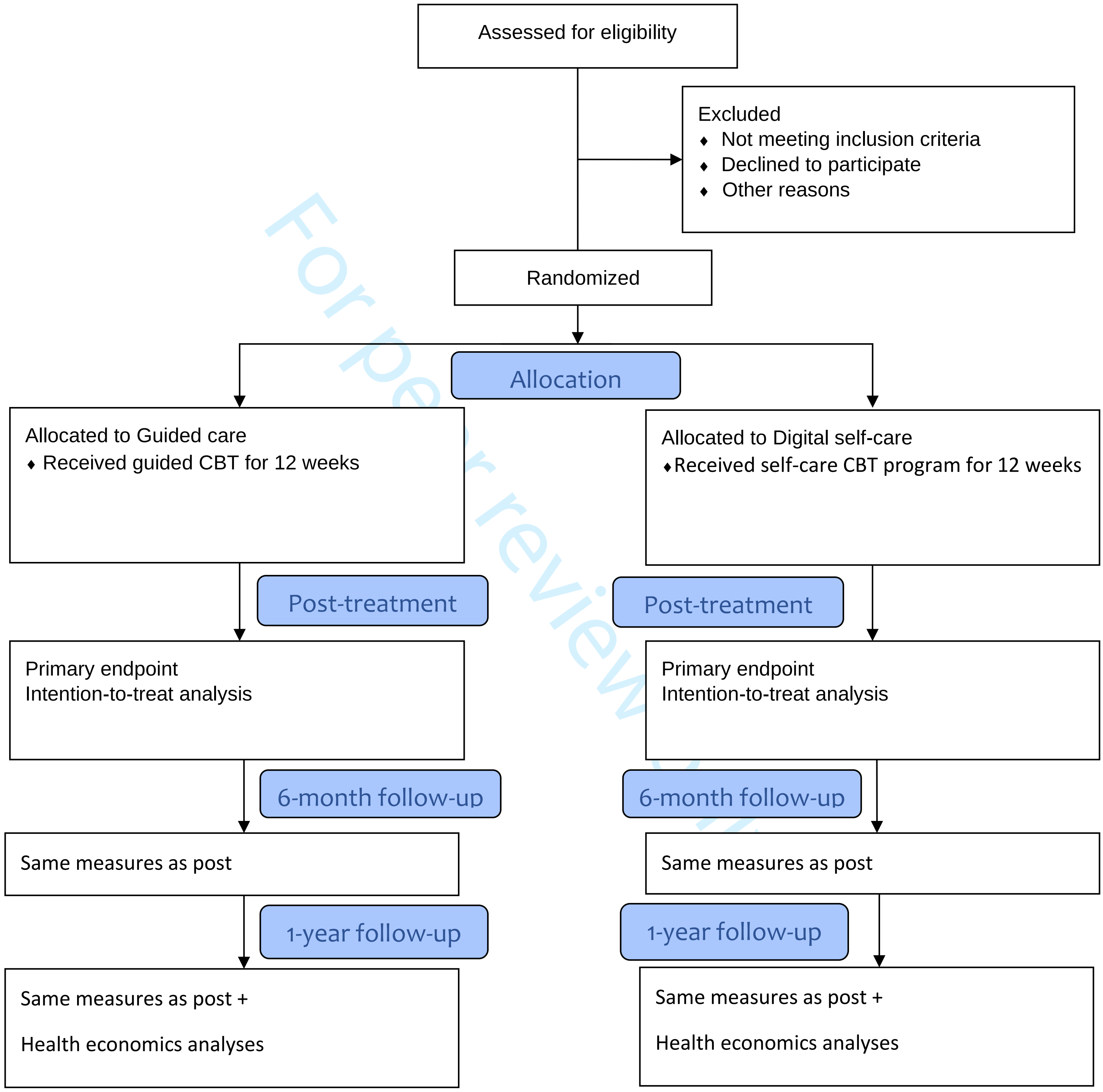

SPIRIT
STANDARD PROTOCOL ITEMS: RECOMMENDATIONS FOR INTERVENTIONAL TRIALS

SPIRIT 2013 Checklist: Recommended items to address in a clinical trial protocol and related documents*

Section/item	Item No	Description	Addressed on page number
**Administrative information**			
Title	1	Descriptive title identifying the study design, population, interventions, and, if applicable, trial acronym	1
Trial registration	2a	Trial identifier and registry name. If not yet registered, name of intended registry	1
	2b	All items from the World Health Organization Trial Registration Data Set	1
Protocol version	3	Date and version identifier	1
Funding	4	Sources and types of financial, material, and other support	14-15
Roles and responsibilities	5a	Names, affiliations, and roles of protocol contributors	1, 14
	5b	Name and contact information for the trial sponsor	N/A (non-pharma. trial)
	5c	Role of study sponsor and funders, if any, in study design; collection, management, analysis, and interpretation of data; writing of the report; and the decision to submit the report for publication, including whether they will have ultimate authority over any of these activities	14-15
	5d	Composition, roles, and responsibilities of the coordinating centre, steering committee, endpoint adjudication committee, data management team, and other individuals or groups overseeing the trial, if applicable (see Item 21a for data monitoring committee)	N/A

For peer review only

Introduction

Background and rationale	6a	Description of research question and justification for undertaking the trial, including summary of relevant studies (published and unpublished) examining benefits and harms for each intervention	3-4
	6b	Explanation for choice of comparators	6-7
Objectives	7	Specific objectives or hypotheses	5
Trial design	8	Description of trial design including type of trial (eg, parallel group, crossover, factorial, single group), allocation ratio, and framework (eg, superiority, equivalence, noninferiority, exploratory)	5

Methods: Participants, interventions, and outcomes

Study setting	9	Description of study settings (eg, community clinic, academic hospital) and list of countries where data will be collected. Reference to where list of study sites can be obtained	6
Eligibility criteria	10	Inclusion and exclusion criteria for participants. If applicable, eligibility criteria for study centres and individuals who will perform the interventions (eg, surgeons, psychotherapists)	6
Interventions	11a	Interventions for each group with sufficient detail to allow replication, including how and when they will be administered	7-8
	11b	Criteria for discontinuing or modifying allocated interventions for a given trial participant (eg, drug dose change in response to harms, participant request, or improving/worsening disease)	N/A
	11c	Strategies to improve adherence to intervention protocols, and any procedures for monitoring adherence (eg, drug tablet return, laboratory tests)	8
	11d	Relevant concomitant care and interventions that are permitted or prohibited during the trial	7
Outcomes	12	Primary, secondary, and other outcomes, including the specific measurement variable (eg, systolic blood pressure), analysis metric (eg, change from baseline, final value, time to event), method of aggregation (eg, median, proportion), and time point for each outcome. Explanation of the clinical relevance of chosen efficacy and harm outcomes is strongly recommended	9-10

Participant timeline	13	Time schedule of enrolment, interventions (including any run-ins and washouts), assessments, and visits for participants. A schematic diagram is highly recommended (see Figure)	Diagram available
Sample size	14	Estimated number of participants needed to achieve study objectives and how it was determined, including clinical and statistical assumptions supporting any sample size calculations	6-7
Recruitment	15	Strategies for achieving adequate participant enrolment to reach target sample size	6

Methods: Assignment of interventions (for controlled trials)

Allocation:

Sequence generation	16a	Method of generating the allocation sequence (eg, computer-generated random numbers), and list of any factors for stratification. To reduce predictability of a random sequence, details of any planned restriction (eg, blocking) should be provided in a separate document that is unavailable to those who enrol participants or assign interventions	9
Allocation concealment mechanism	16b	Mechanism of implementing the allocation sequence (eg, central telephone; sequentially numbered, opaque, sealed envelopes), describing any steps to conceal the sequence until interventions are assigned	9
Implementation	16c	Who will generate the allocation sequence, who will enrol participants, and who will assign participants to interventions	9
Blinding (masking)	17a	Who will be blinded after assignment to interventions (eg, trial participants, care providers, outcome assessors, data analysts), and how	9
	17b	If blinded, circumstances under which unblinding is permissible, and procedure for revealing a participant's allocated intervention during the trial	N/A

Methods: Data collection, management, and analysis

Data collection methods	18a	Plans for assessment and collection of outcome, baseline, and other trial data, including any related processes to promote data quality (eg, duplicate measurements, training of assessors) and a description of study instruments (eg, questionnaires, laboratory tests) along with their reliability and validity, if known. Reference to where data collection forms can be found, if not in the protocol	9-10

For peer review only - http://bmjopen.bmj.com/site/about/guidelines.xhtml

	18b	Plans to promote participant retention and complete follow-up, including list of any outcome data to be collected for participants who discontinue or deviate from intervention protocols	8
Data management	19	Plans for data entry, coding, security, and storage, including any related processes to promote data quality (eg, double data entry; range checks for data values). Reference to where details of data management procedures can be found, if not in the protocol	13
Statistical methods	20a	Statistical methods for analysing primary and secondary outcomes. Reference to where other details of the statistical analysis plan can be found, if not in the protocol	11-12
	20b	Methods for any additional analyses (eg, subgroup and adjusted analyses)	12
	20c	Definition of analysis population relating to protocol non-adherence (eg, as randomised analysis), and any statistical methods to handle missing data (eg, multiple imputation)	12
**Methods: Monitoring**			
Data monitoring	21a	Composition of data monitoring committee (DMC); summary of its role and reporting structure; statement of whether it is independent from the sponsor and competing interests; and reference to where further details about its charter can be found, if not in the protocol. Alternatively, an explanation of why a DMC is not needed	12
	21b	Description of any interim analyses and stopping guidelines, including who will have access to these interim results and make the final decision to terminate the trial	N/A
Harms	22	Plans for collecting, assessing, reporting, and managing solicited and spontaneously reported adverse events and other unintended effects of trial interventions or trial conduct	10
Auditing	23	Frequency and procedures for auditing trial conduct, if any, and whether the process will be independent from investigators and the sponsor	N/A
**Ethics and dissemination**			
Research ethics approval	24	Plans for seeking research ethics committee/institutional review board (REC/IRB) approval	4

Protocol amendments	25	Plans for communicating important protocol modifications (eg, changes to eligibility criteria, outcomes, analyses) to relevant parties (eg, investigators, REC/IRBs, trial participants, trial registries, journals, regulators)	14
Consent or assent	26a	Who will obtain informed consent or assent from potential trial participants or authorised surrogates, and how (see Item 32)	5
	26b	Additional consent provisions for collection and use of participant data and biological specimens in ancillary studies, if applicable	N/A
Confidentiality	27	How personal information about potential and enrolled participants will be collected, shared, and maintained in order to protect confidentiality before, during, and after the trial	12
Declaration of interests	28	Financial and other competing interests for principal investigators for the overall trial and each study site	13
Access to data	29	Statement of who will have access to the final trial dataset, and disclosure of contractual agreements that limit such access for investigators	12
Ancillary and post-trial care	30	Provisions, if any, for ancillary and post-trial care, and for compensation to those who suffer harm from trial participation	9-10
Dissemination policy	31a	Plans for investigators and sponsor to communicate trial results to participants, healthcare professionals, the public, and other relevant groups (eg, via publication, reporting in results databases, or other data sharing arrangements), including any publication restrictions	13
	31b	Authorship eligibility guidelines and any intended use of professional writers	13
	31c	Plans, if any, for granting public access to the full protocol, participant-level dataset, and statistical code	N/A

Appendices

For peer review only - http://bmjopen.bmj.com/site/about/guidelines.xhtml

Informed consent materials	32	Model consent form and other related documentation given to participants and authorised surrogates	Available in Swedish upon request. Could be translated upon reasonable request.
Biological specimens	33	Plans for collection, laboratory evaluation, and storage of biological specimens for genetic or molecular analysis in the current trial and for future use in ancillary studies, if applicable	N/A

*It is strongly recommended that this checklist be read in conjunction with the SPIRIT 2013 Explanation & Elaboration for important clarification on the items. Amendments to the protocol should be tracked and dated. The SPIRIT checklist is copyrighted by the SPIRIT Group under the Creative Commons "Attribution-NonCommercial-NoDerivs 3.0 Unported" license.

For peer review only

BMJ Open

Brief Self-guided Digital Intervention versus a Comprehensive Therapist-guided Online Cognitive Behavioral Therapy for Atopic Dermatitis: A Trial protocol for a Randomized Non-Inferiority Trial

Journal:	*BMJ Open*
Manuscript ID	bmjopen-2022-068908.R2
Article Type:	Protocol
Date Submitted by the Author:	15-Feb-2023
Complete List of Authors:	Kern, Dorian; Karolinska Institutet, Department of Clinical Neuroscience; Stockholm Health Care Services, Department of Clinical Neuroscience<br>Ljótsson, Brjánn; Karolinska Institutet, Department of Clinical Neuroscience, Division of Psychology<br>Lönndahl, Louise; Karolinska Institutet, Department of Medicine Solna<br>Hedman-Lagerlöf, Erik ; Karolinska Institute<br>Bradley, M; Karolinska Institutet<br>Lindefors, Nils; Karolinska Institutet, Department of Clinical Neuroscience<br>Kraepelien, Martin; Karolinska Institutet
**Primary Subject Heading**:	Dermatology
Secondary Subject Heading:	Health economics, Mental health
Keywords:	Eczema < DERMATOLOGY, Health economics < HEALTH SERVICES ADMINISTRATION & MANAGEMENT, Adult dermatology < DERMATOLOGY

SCHOLARONE™
Manuscripts

Trial protocol

Brief Self-guided Digital Intervention versus a Comprehensive Therapist-guided Online Cognitive Behavioral Therapy for Atopic Dermatitis: A Trial protocol for a Randomized Non-Inferiority Trial

Dorian Kern, MSc[1,2]., Brjánn Ljótsson, PhD[2], Louise Lönndahl[3], MD, PhD., Erik Hedman-Lagerlöf[2], PhD[1], Maria Bradley, MD, PhD[3], Nils Lindefors MD, PhD[1], Martin Kraepelien, PhD[1,2]

[1]Centre for Psychiatry Research, Department of Clinical Neuroscience, Karolinska Institutet & Stockholm Health Care Services, Region Stockholm, Norra Stationsgatan 69, SE-113 64 Stockholm, Sweden

[2]Division of Psychology, Department of Clinical Neuroscience, Karolinska Institutet, Nobels väg 9 SE-171 65 Solna, Sweden

[3]Dermatology and Venereology Unit, Department of Medicine Solna, Karolinska Institutet, SE-171 77 Stockholm, Sweden

Correspondence: Dorian Kern; Centre for Psychiatry Research, Norra stationsgatan 69, 113 64, Stockholm; +467 23 24 32 35, dorian.kern@ki.se

Key words: Atopic dermatitis; eczema; pruritus; cognitive behavioral therapy; self-care

Word count: 4417

Version: 1.3

Date: 2023-02-15

For peer review only

Abstract

Introduction Our aim is to investigate whether a shortened digital self-care intervention is noninferior to, and cost-effective compared with, a comprehensive and therapist-guided CBT treatment for AD.

Methods and analysis This is a single-blind, randomized clinical non-inferiority trial at Karolinska Institutet, a medical university in Stockholm, Sweden. We will recruit 174 adult participants with AD through self-referral. Participants will be randomized 1:1 to the two experimental conditions. Participants randomized to guided care will receive internet-delivered cognitive behavior therapy for 12 weeks. Participants randomized to digital self-care will have access to this self-guided intervention for 12 weeks. At post-treatment (primary endpoint), non-inferiority will be tested and resource use will be compared between the two treatment groups. Cost-effectiveness will be explored at 1-year follow-up. Potential mediators will be investigated. Data will be analyzed intention-to-treat. We define non-inferiority as a 3-point difference on the primary outcome measure (Patient-oriented Eczema Measure). Recruitment started in November 2022.

Ethics and dissemination This study is approved by the Swedish ethics authority (reg. no 2021-06704-01) and is preregistered at ClinicalTrials.gov. The study will be reported according to the Consolidated Standards of Reporting Trials statement for non-pharmacological trials. The results of the study will be published in peer-reviewed scientific journals and disseminated to patient organizations and media.

Trial registration: ClinicalTrials.gov identifier: NCT05517850, https://clinicaltrials.gov/ct2/show/NCT05517850

Strengths and limitations of this study

- Includes a full health economics evaluation.
- Broad inclusion criteria will help generalize the results to a variety of AD cases.
- Depending on what time of the year the majority of participants eventually will be recruited, it may be difficult to control for seasonal changes in Sweden's temperate climate.
- Earlier similar studies indicate a risk of bias for the recruitment of female participants with an advanced education.

For peer review only

- If the self-guided condition cannot be found non-inferior, this study cannot answer what specific differences between the conditions caused this result.

Background

Atopic dermatitis

Atopic dermatitis (AD) is the most prevalent of the inflammatory skin diseases. The primary symptoms of AD are itch, dry skin, and rash[1]. Treatment and prevention generally consist of emollient creams, topical corticosteroids, other local immunosuppressive creams such as calcineurin inhibitors. If topical treatment is ineffective, phototherapy and systemic immunomodulatory treatments are often given[2]. In addition to dermatological symptoms, people with AD have an increased risk of major depression and anxiety disorders[3, 4]. Furthermore, behavioral and emotional factors may affect AD symptoms. A common reaction to the itching sensation of the skin is the act of scratching, which, while temporarily relieving, can lead to damage to the skin barrier and an aggravated inflammation[5]. Furthermore, people with AD tend to scratch when they experience anxiety or depressed mood[6], possibly as a conditioned behavior. In addition, avoidance of itch-associated situations is frequently documented, which may lead to avoidance of activities that are otherwise appreciated or important to the patient, which can further affect mental health and quality of life[7] The impairment of quality of life by AD is comparable to asthma and type 1 diabetes[2]. The facts that AD can affect mental health, and that behavior can directly affect AD symptoms, make psychological treatment relevant.

Educational and psychological interventions for atopic dermatitis

Various forms of educational and psychological support may be available for people with AD. According to structured reviews, studies of educational interventions for AD have often had suboptimal methodology and unclear results, with tendencies towards effect on skin-related quality of life and improved symptoms[8]. These interventions may be held in a group or individual setting and may be led by physicians, nurses or other professionals. One recent RCT of an Internet-delivered educational and behavioral

For peer review only

intervention, including young adults, showed small but significant effects on AD symptoms and secondary measures [9].

Internet-delivered cognitive behavioral therapy

Internet cognitive-behavioral therapy (ICBT) is a psychological treatment administered over the Internet, otherwise similar to regular face-to-face CBT. ICBT usually includes therapist support via a secure digital communication, similar to e-mail. Studies have shown that a clinician spends the equivalent of about fifteen minutes per patient and week in ICBT[10]. ICBT has been suggested to improve cost-effectiveness as well as the availability of evidence-based treatment[11] and research reviews show that there are no consistent differences in effectiveness between conventional face-to-face CBT and ICBT[12]. In Sweden, ICBT has been offered in regular care via the Internet Psychiatry Unit in Stockholm since 2007[13].

Although ICBT have many advantages, there is an unmet need for interventions for people with mild to moderate illness, where a comprehensive and therapist-guided ICBT program would be an unnecessarily intense level of care, and thus an inefficient use of resources. One solution is to use existing ICBT programs and simply remove the therapist-guidance. Some research has suggested that this has negative impact on effectiveness, however, there are indications that the amount and type of therapist contact are not decisive[14] and recent studies have compared therapist supported ICBT and self-guided ICBT and shown that treatment outcome, treatment implementation and treatment satisfaction do not differ between conditions if certain components are present in the self-guided intervention. These components include a stringent clinical process in the form of an assessment and a follow-up interview with a clinician and a high-quality interface[15, 16].

Internet-delivered cognitive behavioral therapy for atopic dermatitis

An ICBT protocol has been evaluated for AD. The main components were symptom diary, mindfulness, and exposure to avoided sensations and situations, with prevention of unhelpful responses, such as scrathing. The treatment showed promising preliminary effects in a face-to-face pilot study, which subsequently was confirmed in a randomized

For peer review only

controlled trial (RCT)[17, 18]. A digital self-care intervention has been preliminarily evaluated for people with AD, with promising effects[19]. Digital self-care was based on the ICBT intervention with comparable components. Thus, there is support that CBT can be effective in improving mental health and reducing symptoms of AD, although additional studies are needed. A comparison between the ICBT and reworked digital self-care variants could further contribute to the literature and the results will have implications for implementation.

This is a study protocol of a single-blind non-inferiority RCT, aimed at evaluating the effects of a self-guided CBT program available via the internet (digital self-care) for people with AD, in relation to a previously evaluated therapist-guided internet-delivered CBT program (guided care)[18]. The main hypothesis is that digital self-care will be non-inferior to guided care in reducing self-rated eczemic symptoms, as measured by the Patient-oriented eczema measure (POEM)[20]. Secondary purposes are to evaluate use of resources and cost-effectiveness of digital self-care compared to guided care, and to investigate potential mediators of symptom improvement.

Hypotheses

Primary hypothesis: Digital self-care will be non-inferior to ICBT, according to the predefined non-inferiority margin of 3 points, as measured by the primary outcome (POEM).

Secondary hypothesis 1: Digital self-care will be associated with significantly less staff costs than ICBT, in the form of time spent in hours by a licensed psychologist.

Secondary hypothesis 2: Digital self-care will be cost-effective compared to ICBT, assessed at one-year follow-up.

Secondary hypothesis 3: Effects on the outcome will be mediated by the amount of time spent by participants on main treatment components.

For peer review only

Study design

Timeframe

The first participant was included 2022-29-11. The first cohort started 2022-05-12. Post treatment data collection is expected to finish summer or autumn 2023, with the last follow-up a year after that. The present study is a non-inferiority RCT for adults with AD. Participants randomized to guided care will receive ICBT via the internet, guided by a therapist for 12 weeks. Participants randomized to digital self-care get access to a self-guided CBT-based intervention for 12 weeks. Condition allocation will be 50/50.

The primary end point is at 12 weeks, (i.e., post-treatment). The study will also include a six-month and a one-year follow-up. 1 year after treatment conclusion, long-term effects will be evaluated. An overview of the trial design can be seen in Figure 1.

The study is approved by the Swedish ethics authority (reg. no 2021-06704-01) and is preregistered at ClinicalTrials.gov. The participants will receive written and verbal information about the study. The participants will digitally sign an informed consent in a secure platform.

Participants

Participants will be recruited via advertisements in social media and with the help of relevant patient associations' mailing lists and websites. Interested individuals with self-reported AD diagnosis may participate following their written consent to the study. Advertisements in different types of social media has the advantage that it reaches different types of people regarding age and gender. In some platforms, it is possible to make the advertisements to only appear for men, if the female percentage should be considered too high, which is something we could consider. In order to minimize false diagnoses, participants will disclose at which point in time they received their AD diagnosis, and if they can recall, at what clinic and by which physician. They will then receive login information to access a secure digital platform, where data from self-assessment scales will be registered. Participation in the study does not involve any change in their regular treatment or medical self-care. Participants will be evaluated by a licensed psychologist in a clinical interview, assessing suitability according to the

For peer review only

inclusion criteria and motivation. If a potential participant states that they are interested, knowing that these interventions will require considerable time and effort, that participant is considered motivated.

Inclusion criteria: At least 18 years of age with self-reported AD diagnosis. No new types of medications introduced for six months prior, with no intention of future change (participants will not be excluded if they change their medication after inclusion). Participants need to be able to understand written and spoken Swedish, have access to the internet and be able to receive telephone calls and text messages.

Exclusion criteria: Other disease or condition that has immediate treatment priority over AD. This could be an ongoing demanding treatment of a severe somatic or psychiatric condition. No specific diagnoses are inevitably a cause for exclusion, as potential participants will rather be evaluated on a case-to-case basis.

Setting

The trial is conducted at Karolinska Institutet, a medical university in Stockholm, Sweden. Dermatologists involved in the study are also associated with the dermatology clinic at Karolinska University Hospital, in Stockholm, Sweden. Initial assessment will be made by telephone by a clinical psychologist, and both study arms are entirely conducted online, including the collection of self-rated assessments. Participants will be recruited from all parts of Sweden.

Power calculation

Based on data from Hedman-Lagerlöf and colleagues[17] and Kern and colleagues[18] we expect an in-group effect of about d=0.85 for both interventions. A consultant statistician performed a power calculation. The required sample size was computed based on boot-strapped (5000 simulations) reruns of weekly measured data obtained in the therapist-guided RCT[17]. The results indicated that a sample size of 174 would be required for 90% power, α=.05, given a non-inferiority margin of 3 points on the POEM scale and accounting for 10% missing data.

For peer review only

Planned interventions

Usual care

Participants will continue their usual care. According to national guidelines, people with AD should be treated according to a stepped-care model, with emollients and education at the bottom, advancing to cortisone treatments, up to more advanced treatments, such as immunosuppressive and biological interventions. Between individuals, usual care in Sweden can vary greatly. People with AD in Sweden may be treated and followed up at primary or specialist levels. Not all people with AD have regular contact with a physician at all, some have intermittent check-ups, and others may have close and frequent contact. Psychological interventions are not usually available, whereas educational interventions may be available in some regions of Sweden.

Guided care

The aim of CBT for AD is to target behavioral and emotional processes, to improve AD symptoms and increase quality of life. The participants read education material, and use worksheets and homework reports. The material is in Swedish and is currently only available to study personnel and participants. This intervention consists of 10 modules and lasts 12 weeks. An introduction provides information about CBT and AD. Participant register itching and scratching behaviors, with the aim to increase awareness of these behaviors. Gradually, the participants are introduced to the proposed active treatment components: mindfulness training and exposure with response prevention. Mindfulness is trained throughout the intervention. The purpose of mindfulness is to increase awareness of emotional and behavioral reaction patterns, and to increase tolerance of aversive inner experiences[21]. Additionally, mindfulness training may improve stress tolerance. Participants are also trained in self-compassion, to increase understanding of one's own reactions and to increase the ability to handle difficult situations in the treatment and life[22]. After practicing these fundamental skills, exposure with response prevention is introduced in week 4. Exposure is a controlled way to gradually approach situations that evoke strong emotions or somatic reactions. The purpose is to gain knowledge and experience in how this approach can change one's perception of unwanted situations, emotions, or somatic reactions[23].

For peer review only

The assumption is that skills learned from mindfulness practice and self-compassion training helps the patient to conduct exposure exercises. Response prevention means to reduce scratching in these problematic situations in order to reduce itch in the long term. Through this whole process, the participant is guided by a therapist. The therapist may be contacted through a secure internet platform at any time and responds within 48 hours. Participants read the education material, construct exercises to perform, describe their progress in homework assignments, and receive feedback from their therapist. During the 12-week period, the participants work at their own pace, and contacts their therapist when necessary. The recommended pace, however, is one module per week, and the therapist contact participants who are inactive. All participants have access to the same treatment material but will construct exposure exercises that fit their specific situation, assisted by the therapist. The therapists will guide participants to make exercises as faithful to the treatment as possible. Participants will report back what they have done. Because therapist and participants never meet, there is no control of whether participants actually perform exercises as they are reported. The study coordinator will offer guidance to other therapists if needed.

Digital self-care

As with guided care, the aim of CBT for AD is to target behavioral and emotional processes, to improve AD symptoms and increase quality of life. The material is similar to guided care but shortened. Likewise, participants, read information, and perform exercises in their everyday life. As with guided care, the main treatment components are mindfulness and exposure with response prevention, with a specific focus on itch. Participants will be able to practice mindfulness with the help of instructions and examples. Because it may be challenging to construct relevant exposure exercises without the help of a therapist, participants will receive detailed instructions and guidance, and get to choose exposure exercises from a long list of examples. They will also be free to create their own relevant exposure situations within the intervention. Digital self-care consists of 8 modules. The only active interventions, mindfulness and exposure are introduced week 1 and 2, respectively. From week 3-7, no new interventions are introduced and participants are advised to continue practicing the interventions, but there is a new theme of education each week, to act as inspiration for exercises. The last 4 weeks, the participant is merely advised to continue practice. There

For peer review only

is no therapist, but the study coordinator monitors the process and may contact inactive participants. Participants will access the same digital platform as the guided condition. Participants work entirely at their own pace. Modules will be unlocked automatically if one week has passed, and all material in the previous module has been read. Participants will report if they have done their assignments, but there is no control if they do as they report.

Differences between study conditions

The main differences between the conditions are that digital self-care a) has contact with a clinician at only two points in time: pre-intervention and after eight weeks plus the addition of automated messages, whereas guided care has continuous contact throughout the treatment via text messages on a secure platform, and that b) the treatment text is significantly shortened. Automated emails are sent every week to the non-guided group, consisting of a greeting, and some encouraging words relevant to the week's content. Both groups receive automated text messages at the time of new assessments. Guided care will also have an evaluation from a clinician via telephone at two points in time. Furthermore, digital self-care uses a mobile optimized interface, and have improvements in design aimed to decrease the need of guidance.

Therapists

Only the guided group will have therapist contact. The self-guided group will be managed by the study coordinator. The therapists are licensed psychologists specialized in CBT. All therapists have experience in working with somatic conditions and in a text-based format. All therapists will participate in an introductory workshop before treating in the study. The project coordinator will supervise the therapists regularly and on-demand.

Randomization/blinding

Participants will be blinded to condition. They will be generally informed that they will be randomized to "either one of two types of self-help interventions based on CBT", not knowing any difference between the conditions. After baseline measurements, included participants will be allocated randomly (1:1) to therapist-guided or self-guided treatment in consecutive even-numbered blocks. Randomization will be based on a true

For peer review only

random number service and conducted by a person blinded to the participants, who is not otherwise involved with the study, using a true random number generator (http://www. random.org). It is impossible for the psychologist performing the inclusion assessment to anticipate participant allocation.

Measurements

The outcome measures are based on those used previously by Hedman-Lagerlöf and colleagues[17] and Kern and colleagues[18], for the evaluation of CBT interventions for AD. The battery of instruments in the current study overlaps with the recently published HOME criteria (harmonizing measures for eczema), but have several additional measures, and does not include some of the suggested measures [24]. We do include the suggested measurements for self-reported symptoms and quality of life but have no clinician-rated measurement of symptoms, which we acknowledge as a weakness.

Primary outcome measure: Patient Oriented Eczema Measure (POEM)[20] measures the severity of symptoms of atopic dermatitis, which has a range of 0 to 28. The POEM has excellent psychometric properties and is widely used[25]. In this study, in order to be conservative, response is operationalized as a POEM score reduction 4 points or more, and the non-inferiority margin is operationalized as 3 points. This is the same as earlier studies of CBT for AD, based on changes from severe eczema. Another evaluation has found smaller changes, around 2.1 to 2.9 as "likely to be beyond measurement error but unlikely to be clinically important"[26]. Because of this, as we are including participants with mild, moderate and severe eczema, we could possibly have chosen an even smaller margin.

Secondary outcome measures: Demographical data will be collected upon screening. Peak pruritus numerical rating scale (NRS)[27], is used to measure itching sensations; Perceived Stress Scale (PSS)[28] is used to measure general experience of stress; Patient Health Questionnaire-9 (PHQ-9)[29] is used to measure depressive symptoms; Dermatological Life Quality Index (DLQI)[30] is used to measure AD-specific quality of life; Insomnia severity index (ISI)[31] is used to measure severity of sleep problems and possible insomnia; Client Satisfaction Questionnaire (CSQ-8)[32] is used to measure how satisfied participants are with the intervention. We will also ask participants to report if they experience adverse events associated with the treatment (AE). In addition

For peer review only

to this, we will use the Credibility / Expectancy Scale (CES)[33] to measure credibility. At the end of the intervention, the participants will be asked to estimate the Time spent in the Exercises (ToE). Euro-QOL 5 dimensions (EQ-5D)[34] will be used to assess quality of life from a health economics perspective. Treatment Inventory of Costs in Psychiatric Patients (TIC-P)[35] will be used to assess health-care costs. Participant will also rate their user experience of the interventions with the System Usability Scale (SUS) [36]. Resource use is measured by the time in minutes the clinician spends on the different tasks associated with administering the intervention, doing interviews, and giving support, or Time spent on Treating (ToT). Therapists will register their time spent throughout the intervention, and not at a specific measurement point.

Outcomes at all scales will be compared with available published results regarding other Internet-based interventions for each scale.

Measurement points

Please refer to table 1. There are no planned interim analyses. There are no predefined criteria for trial termination.

Table 1. Measurement points

**Screening**	**Pre**	**Weekly**	**Week2**	**MID**	**POST**	**FU6**	**FU12**
Demo	POEM	POEM	CES	POEM	POEM	POEM	POEM
POEM	NRS	NRS		NRS	NRS	NRS	NRS
NRS	DLQI	DLQI		DLQI	DLQI	DLQI	DLQI
DLQI	PHQ-9	PHQ-9		PHQ-9	PHQ-9	PHQ-9	PHQ-9
PHQ-9	PSS	PSS		PSS	PSS	PSS	PSS
	ISI	ISI		TIC-P	TIC-P	TIC-P	TIC-P
	TIC-P			EQ-5D	EQ-5D	EQ-5D	EQ-5D
	EQ-5D			ISI	ISI	ISI	ISI
					CSQ-8		
					AE		

For peer review only

ToE

SUS

Demo = demographical data; POEM = Patient Oriented Eczema Measure; NRS = Peak Pruritus Numerical Rating Scale; DLQI = Dermatological Life Quality Index; PHQ-9 = Patient Health Questionnaire 9 item; PSS = Perceived Stress Scale; ISI = Insomnia Severity Index; TIC-P = Treatment Inventory of Costs in Psychiatric Patients; EQ-5D = Euro-QOL 5 dimensions; CES = Credibility/Expectancy Scale; AE = Adverse Events; ToE = Time (spent) on Exercises SUS = System Usability Scale

Safety considerations

Participants' regular healthcare is unaffected by either of the study conditions. Possible benefits for patients with increased ability to manage their disease are presumed to justify any disadvantages in the form of time consumption and temporary discomfort. Adverse treatment effects will be examined after the total twelve weeks of the intervention, and will be acted on if they are spontaneously reported before. If the disease worsens, the participants will be encouraged to seek help from their regular specialist clinic or primary health care center. All communication and data storage take place on an encrypted and secure digital platform and is in accordance with the Karolinska Institutet's regulations and the EU General Data Protection Regulation. In case of any serious harm, the standard patient's insurance for Government financed health care will be applicable to every participant.

Statistical analysis

Non-inferiority margin

Earlier research[22] has defined clinically significant improvement on the main outcome measure to be 3,4 points, which is the basis for our non-inferiority margin of 3 points.

For peer review only

Non-inferiority

Change over time will be modelled using mixed-effects linear regression with a random intercept and slope (time). The main outcome POEM will be analyzed by the regression on time (baseline to end point), group (digital self-care vs guided care), and the interaction of time and group. We will consider digital care to be non-inferior to therapist-guided if the upper bound of the 1-sided 95% confidence interval for the time × group interaction ($\alpha = .05$) is within the a-priori non-inferiority margin of 3 points on the POEM[36]. In addition, we will investigate non-inferiority in a sensitivity analysis per-protocol framework with data from patients who will have initiated module 4 or further in either of the study arms. As for interpretation of results, if the two-sided 95% confidence interval does not contain zero, this will be viewed as indicative of superiority of the corresponding treatment. We will estimate Cohen's *d* by dividing the time × group coefficient by the pooled observed baseline standard deviation. All primary analyses will be conducted intent-to-treat. The power calculations allow for 10% missing post-treatment data on the primary outcome at 90% power. If we have more than 10% missing data, we will use multiple imputation before conducting the primary analysis.

Resource utilization

At the primary endpoint, resource utilization will be preliminary assessed by measuring therapist time for each treatment condition and compared with a typical salary for a clinical psychologist employed in Stockholm, Sweden.

Cost-effectiveness

At one-year follow-up, a cost-effectiveness analysis will be performed from a-healthcare system perspective, based on recorded therapist time, as well as from a societal perspective. The incremental cost-effectiveness ratio will be estimated at post-treatment and at follow-up. We will also use the TIC-P to collect economic cost data[34], together with the EQ-5D[35]. The costs will be assessed in Swedish krona and converted into US dollar using 2023 as the reference year. The costs of the self-guided and therapist-guided treatments will be modelled as a function of therapist time using the same healthcare index to determine tariffs for licensed psychologists. According to our hypothesis, there should be a significant difference in treatment costs but there

For peer review only

should not be any significant difference in societal costs. We will calculate quality-adjusted life-years using the area under the curve approach[37], use regression analyses to analyze costs[38], and use nonparametric bootstrapping with 5000 iterations, where differences in costs and outcomes will be paired. The incremental cost-effectiveness ratio will be used as a cost-effectiveness estimate[39].

Mediation

We are interested in the mediating effect of time spent on mindfulness and exposure exercises on the outcome. Because we have a non-inferiority hypothesis, we will not perform traditional mediation analysis where the mediator is proposed to account for between-group differences. Instead, we will do within-group process analysis to investigate if treatment activity predicts simultaneous and/or subsequent change in POEM during treatment[40]. The exact analytic model will be determined based on the nature of data that we are able to collect on treatment activity. We will also explore potential moderated mediation, i.e., if mediators differ between the two types of treatment. We also intend to analyze self-rated pruritus, perceived stress, and depressed mood as potential mediators.

Data

All data will be stored at a secure digital platform, to which only study personnel have access. Measurements from the participants will be submitted in the platform itself. Because all data is submitted and stored in the same digital platform, we will not use a data monitoring committee. The final trial data set will be primarily handled by the first, second and final author. However, all authors will be granted access to the data set if necessary. We have no plans to make an individual-level data set or any statistical code public. Data will be available upon reasonable request to the corresponding author.

Patient and public involvement

We have used input from participants in the feasibility trial[19], on which we based intervention improvements in the present study. This included various revisions, the most important of which was to add a “scratch-free time” intervention. The duration is defined by the participant, and gradually increased. This ensured an easier start

For peer review only

compared to the previous variant to begin with an entire scratch-free day. Some participants also wanted therapist guidance, which could not be considered for this particular intervention. We also considered feedback from a patient representative, who was not a trial participant. They largely agreed with the feedback from trial participants, encouraged the intervention in its current form, and furthermore participated in an introductory seminar with the treating study psychologists. In the current trial, no participants were involved in decisions about study design, outcome measures, or research question. A patient organization for AD, as well as one for disorders related to asthma and allergies (Atopikerna and Astma & Allergiförbundet) will be involved in the recruitment of participants by informing their members about the study. We plan to disseminate the results of the research to study participants and to the aforementioned patient organizations.

Ethics and Dissemination

The study will be carried out according to the predefined standards of this trial protocol, the Declaration of Helsinki and Good Clinical Practice. This study will be reported according to the CONSORT statement for non-pharmacological trials[41]. The interventions that will be administered have deemed safe by consultant dermatologists and have been previously evaluated without serious adverse events[18,19]. This study will use the Vancouver criteria for authorship. The results of this study will be published in peer-reviewed scientific journals, be presented to the scientific community at conferences. The results will also be communicated to study participants, patient organizations and clinicians.

Acknowledgement

Author contributions

Dorian Kern: Study design, intervention design, drafting of manuscript, planning of statistical analysis. Brjánn Ljótsson: Study design, intervention design, planning of statistical analysis. Louise Lönndahl: Contributions to intervention and manuscript; Maria Bradley: Study concept and contributions to manuscript. Erik Hedman-Lagerlöf: Study concept, contributions to manuscript and planning of statistical analysis; Nils

For peer review only

Lindefors: Study concept, study design, contributions to manuscript. Martin Kraepelien: Study concept, contributions to manuscript, intervention design, study design, planning of statistical analysis, principal investigator. All authors: Critical review and revision of manuscript.

Funding/support

This study will be funded in part by the Swedish Ministry of Health and Social affairs. The funder has no role in the design and conduct of the study and will have no influence of data collection, management, analysis, interpretation. The funder will have no part in preparation, review or approval of the manuscript, or the decision to submit the manuscript for publication.

Declaration of conflict of interest

Dr Ljótsson reported being a shareholder of DahliaQomit AB and holding copyright for a cognitive behavioral treatment manual for irritable bowel syndrome, with royalties paid from Pear Therapeutic, Inc, outside of submitted work. Dr Lönndahl reported receiving a personal fee from LEO Pharma and Sanofi Genzyme outside of submitted work. Dr Hedman-Lagerlöf reported being a shareholder of DahliaQomit AB and holding copyright for a cognitive behavioral treatment manual for irritable bowel syndrome, with royalties paid from Pear Therapeutic, Inc, outside of submitted work. No other disclosures were reported.

References

1. Drucker AM, Wang AR, Li WQ, et al. The Burden of Atopic Dermatitis: Summary of a Report for the National Eczema Association. *J Invest Dermatol*. 2017;137(1):26-30. doi:10.1016/j.jid.2016.07.012
2. Weidinger S & Novak N. Atopic dermatitis. *Lancet*. 2016;387(10023):1109-1122. doi:10.1016/S0140-6736(15)00149-X
3. Yaghmaie P, Koudelka CW, Simpson EL. Mental health comorbidity in patients with atopic dermatitis. *J Allergy Clin Immunol.* 2013; 131(2): 428-33

For peer review only

4. Sandhu JK et al, Wu KK, Bui T-L, Armstrong AW. Association Between Atopic Dermatitis and Suicidality: A Systematic Review and Meta-analysis. *JAMA Dermatology*. 2019; 155(2): 178-87.
5. Wahlgren CF. Itch and atopic dermatitis: an overview. *J Dermatol.* 1999; 26(11): 770-9
6. Daunton A, Bridgett C, Goulding JM. Habit reversal for refractory atopic dermatitis: a review. *Br J Dermatol.* 2016; 174(3): 657-9.
7. Verhoeven L, Kraaimaat F, Duller P, et al. Cognitive, behavioral, and physiological reactivity to chronic itching: analogies to chronic pain. *International journal of behavioral medicine.* 2006; 13(3): 237-43.
8. Pickett K, Loveman E, Kalita N, Frampton G, Jones J. Educational interventions to improve quality of life in people with chronic inflammatory skin diseases: systematic reviews of clinical effectiveness and cost-effectiveness. *Health Technology Assessment*. 2015;19(86):i-176.
9. Santer M, Muller I, Becque T, Stuart B, Hooper J, Steele M et al. Eczema Care Online behavioural interventions to support self-care for children and young people: two independent, pragmatic, randomised controlled trials *BMJ* 2022; 379 :e072007 doi:10.1136/bmj-2022-072007
10. Hedman E, Ljótsson B, Kaldo V, et al. Effectiveness of Internet-based cognitive behaviour therapy for depression in routine psychiatric care. *J Affect Disord*. 2014;155:49-58. doi:10.1016/j.jad.2013.10.023
11. Cavanagh K. Geographic inequity in the availability of cognitive behavioural therapy in England and Wales: a 10-year update. *Behav Cogn Psychother*. 2014;42(4):497-501. doi:10.1017/S1352465813000568
12. Carlbring P, Andersson G, Cuijpers P, et al. Internet-based vs. face-to-face cognitive behavior therapy for psychiatric and somatic disorders: an updated systematic review and meta-analysis. *Cogn Behav Ther*. 2018;47(1):1-18. doi:10.1080/16506073.2017.1401115
13. Lindefors N, El Alaoui S, Svanborg C. Internet-KBT ger effektiv behandling - Signifikant hållbar förbättring vid depression, paniksyndrom och socialt ångestsyndrom [Internet CBT provides effective treatment]. *Läkartidningen*. 2017;114:D77R. Published 2017 Jan 24.

For peer review

14. Hirai M, Clum GA. A meta-analytic study of self-help interventions for anxiety problems. *Behav Ther*. 2006;37(2):99-111. doi:10.1016/j.beth.2005.05.002
15. Dear BF, Staples LG, Terides MD, et al. Transdiagnostic versus disorder-specific and clinician-guided versus self-guided internet-delivered treatment for Social Anxiety Disorder and comorbid disorders: A randomized controlled trial. *J Anxiety Disord*. 2016;42:30-44. doi:10.1016/j.janxdis.2016.05.004
16. Titov N, Dear BF, Staples LG, et al. Disorder-specific versus transdiagnostic and clinician-guided versus self-guided treatment for major depressive disorder and comorbid anxiety disorders: A randomized controlled trial. *J Anxiety Disord*. 2015;35:88-102. doi:10.1016/j.janxdis.2015.08.002
17. Hedman-Lagerlöf E, Bergman A, Lindefors N, et al.. Exposure-based cognitive behavior therapy for atopic dermatitis: an open trial. *Cogn Behav Ther*. 2019;48(4):300-310. doi:10.1080/16506073.2018.1504320
18. Hedman-Lagerlöf E, Fust J, Axelsson E, et al. Internet-Delivered Cognitive Behavior Therapy for Atopic Dermatitis: A Randomized Clinical Trial. *JAMA Dermatol*. 2021;157(7):796-804. doi:10.1001/jamadermatol.2021.1450
19. Kern D, Ljótsson B, Lönndahl L, et al. Optimized User Experience, Efficiency, and Resource Use in Online Self-Management of Atopic Dermatitis. *JAMA Dermatol.* 2022;158(11):1325–1327. doi:10.1001/jamadermatol.2022.3434
20. Charman CR, Venn AJ, Williams HC. The Patient-Oriented Eczema Measure: development and initial validation of a new tool for measuring atopic eczema severity from the patients' perspective. *Arch Dermatol* 2004; 140:1513–19.
21. Hayes SC, Luoma JB, Bond FW, et al,. Acceptance and commitment therapy: model, processes and outcomes. *Behav Res Ther*. 2006;44(1):1-25. doi:10.1016/j.brat.2005.06.006
22. Clarke EN, Thompson AR, Norman P. Depression in people with skin conditions: The effects of disgust and self-compassion. *Br J Health Psychol*. 2020;25(3):540-557. doi:10.1111/bjhp.12421
23. Sewart AR, Craske MG. Inhibitory learning. In: Abramowitz JS, Blakey SM, editors. Clinical handbook of fear and anxiety: Maintenance processes and treatment mechanisms. Washington, DC, USA: American Psychological Association; 2020:265-285.

24. Williams HC, Schmitt J, Thomas KS, et al. The HOME Core outcome set for clinical trials of atopic dermatitis. *J Allergy Clin Immunol*. 2022;149(6):1899-1911. doi:10.1016/j.jaci.2022.03.017
25. Schram ME, Spuls PI, Leeflang MM, et al. EASI, (objective) SCORAD and POEM for atopic eczema: responsiveness and minimal clinically important difference. *Allergy*. 2012;67(1):99-106. doi:10.1111/j.1398-9995.2011.02719.x
26. Howells L, Ratib S, Chalmers JR, Bradshaw L, Thomas KS, CLOTHES trial team. How should minimally important change scores for the Patient-Oriented Eczema Measure be interpreted? A validation using varied methods. *British Journal of Dermatology.* 2018 May;178(5):1135-42.
27. Yosipovitch G, Reaney M, Mastey V, et al. Peak Pruritus Numerical Rating Scale: psychometric validation and responder definition for assessing itch in moderate-to-severe atopic dermatitis. *Br J Dermatol*. 2019;181(4):761-769. doi:10.1111/bjd.17744
28. Cohen S, Kamarck T, Mermelstein R. A global measure of perceived stress. *J Health Soc Behav*. 1983;24(4):385-396.
29. Kroenke K, Spitzer RL, Williams JB. The PHQ-9: validity of a brief depression severity measure. *J Gen Intern Med.* 2001; 16(9): 606-13.
30. Finlay AY, Khan GK. Dermatology Life Quality Index (DLQI)--a simple practical measure for routine clinical use. *Clin Exp Dermatol.* 1994; 19(3): 210-6.
31. Morin, C.M. Insomnia: Psychological Assessment and Management. Guilford, New York; 1993
32. Attkisson CC, Zwick R. The client satisfaction questionnaire. Psychometric properties and correlations with service utilization and psychotherapy outcome. *Eval Program Plann*. 1982;5(3):233-237. doi:10.1016/0149-7189(82)90074-x
33. Devilly GJ, Borkovec TD. Psychometric properties of the credibility/expectancy questionnaire. *J Behav Ther Exp Psychiatry*. 2000;31(2):73-86. doi:10.1016/s0005-7916(00)00012-4
34. Bouwmans C, De Jong K, Timman R, et al. . Feasibility, reliability and validity of a questionnaire on healthcare consumption and productivity loss in patients with a psychiatric disorder (TiC-P). BMC Health Serv Res. 2013;13(1):217. doi:10.1186/1472-6963-13-217 - DOI - PMC - PubMed

35. Rabin R, de Charro F. EQ-5D: a measure of health status from the EuroQol Group. *Ann Med*. 2001;33(5):337-343. doi:10.3109/07853890109002087
36. Lewis J. System Usability Scale: Past Present and Future. *INT J HUM-COMPUT INT.* 2018:34(7): 577-590.
37. Walker J. Non-inferiority statistics and equivalence studies. *BJA Educ*. 2019;19(8):267-271. doi:10.1016/j.bjae.2019.03.004
38. Matthews JNS, Altman DG, Campbell MJ, et al. Analysis of serial measurements in medical research. BMJ. 1990;300(6719):230-235. doi:10.1136/bmj.300.6719.230 - DOI - PMC - PubMed
39. Manning WG, Mullahy J. Estimating log models: to transform or not to transform?. *J Health Econ*. 2001;20(4):461-494. doi:10.1016/s0167-6296(01)00086-8
40. H. Hesser. Modeling individual differences in randomized experiments using growth models: recommendations for design, statistical analysis and reporting of results of internet interventions Internet Interv. 2015, 110-120, 10.1016/j.invent.2015.02.003
41. Schulz KF , Altman DG , Moher D . CONSORT 2010 Statement: updated guidelines for reporting parallel group randomised trials. *BMC Med* 2010;8:18.doi:10.1186/1741-7015-8-18

Figure 1 CONSORT 2010 Flow Diagram

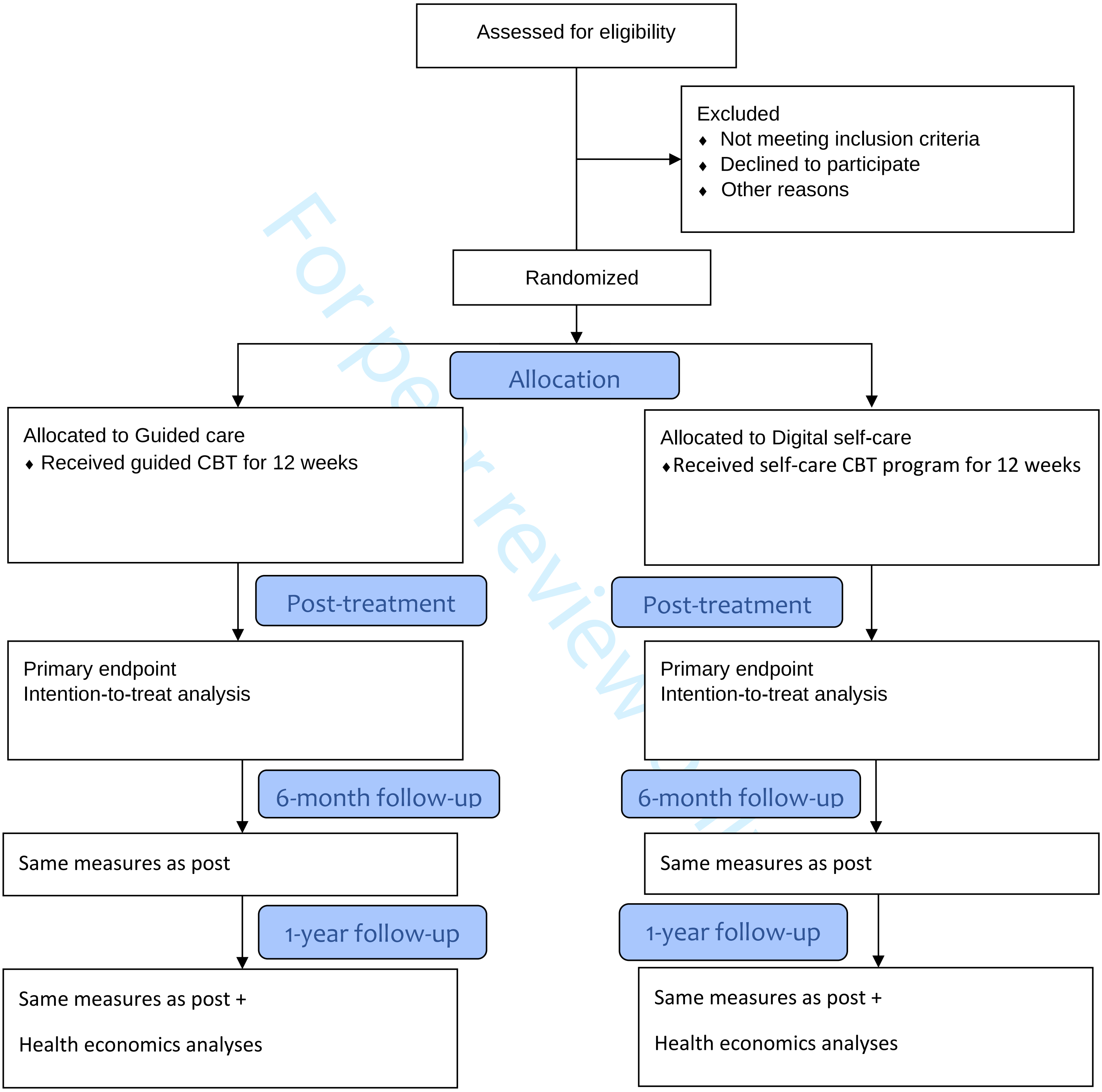

SPIRIT
STANDARD PROTOCOL ITEMS: RECOMMENDATIONS FOR INTERVENTIONAL TRIALS

SPIRIT 2013 Checklist: Recommended items to address in a clinical trial protocol and related documents*

Section/item	Item No	Description	Addressed on page number
**Administrative information**			
Title	1	Descriptive title identifying the study design, population, interventions, and, if applicable, trial acronym	1
Trial registration	2a	Trial identifier and registry name. If not yet registered, name of intended registry	1
	2b	All items from the World Health Organization Trial Registration Data Set	1
Protocol version	3	Date and version identifier	1
Funding	4	Sources and types of financial, material, and other support	14-15
Roles and responsibilities	5a	Names, affiliations, and roles of protocol contributors	1, 14
	5b	Name and contact information for the trial sponsor	N/A (non-pharma. trial)
	5c	Role of study sponsor and funders, if any, in study design; collection, management, analysis, and interpretation of data; writing of the report; and the decision to submit the report for publication, including whether they will have ultimate authority over any of these activities	14-15
	5d	Composition, roles, and responsibilities of the coordinating centre, steering committee, endpoint adjudication committee, data management team, and other individuals or groups overseeing the trial, if applicable (see Item 21a for data monitoring committee)	N/A

For peer review only

**Introduction**			
Background and rationale	6a	Description of research question and justification for undertaking the trial, including summary of relevant studies (published and unpublished) examining benefits and harms for each intervention	3-4
	6b	Explanation for choice of comparators	6-7
Objectives	7	Specific objectives or hypotheses	5
Trial design	8	Description of trial design including type of trial (eg, parallel group, crossover, factorial, single group), allocation ratio, and framework (eg, superiority, equivalence, noninferiority, exploratory)	5
**Methods: Participants, interventions, and outcomes**			
Study setting	9	Description of study settings (eg, community clinic, academic hospital) and list of countries where data will be collected. Reference to where list of study sites can be obtained	6
Eligibility criteria	10	Inclusion and exclusion criteria for participants. If applicable, eligibility criteria for study centres and individuals who will perform the interventions (eg, surgeons, psychotherapists)	6
Interventions	11a	Interventions for each group with sufficient detail to allow replication, including how and when they will be administered	7-8
	11b	Criteria for discontinuing or modifying allocated interventions for a given trial participant (eg, drug dose change in response to harms, participant request, or improving/worsening disease)	N/A
	11c	Strategies to improve adherence to intervention protocols, and any procedures for monitoring adherence (eg, drug tablet return, laboratory tests)	8
	11d	Relevant concomitant care and interventions that are permitted or prohibited during the trial	7
Outcomes	12	Primary, secondary, and other outcomes, including the specific measurement variable (eg, systolic blood pressure), analysis metric (eg, change from baseline, final value, time to event), method of aggregation (eg, median, proportion), and time point for each outcome. Explanation of the clinical relevance of chosen efficacy and harm outcomes is strongly recommended	9-10

For peer review only - http://bmjopen.bmj.com/site/about/guidelines.xhtml

Participant timeline	13	Time schedule of enrolment, interventions (including any run-ins and washouts), assessments, and visits for participants. A schematic diagram is highly recommended (see Figure)	Diagram available
Sample size	14	Estimated number of participants needed to achieve study objectives and how it was determined, including clinical and statistical assumptions supporting any sample size calculations	6-7
Recruitment	15	Strategies for achieving adequate participant enrolment to reach target sample size	6
**Methods: Assignment of interventions (for controlled trials)**			
Allocation:			
Sequence generation	16a	Method of generating the allocation sequence (eg, computer-generated random numbers), and list of any factors for stratification. To reduce predictability of a random sequence, details of any planned restriction (eg, blocking) should be provided in a separate document that is unavailable to those who enrol participants or assign interventions	9
Allocation concealment mechanism	16b	Mechanism of implementing the allocation sequence (eg, central telephone; sequentially numbered, opaque, sealed envelopes), describing any steps to conceal the sequence until interventions are assigned	9
Implementation	16c	Who will generate the allocation sequence, who will enrol participants, and who will assign participants to interventions	9
Blinding (masking)	17a	Who will be blinded after assignment to interventions (eg, trial participants, care providers, outcome assessors, data analysts), and how	9
	17b	If blinded, circumstances under which unblinding is permissible, and procedure for revealing a participant's allocated intervention during the trial	N/A
**Methods: Data collection, management, and analysis**			
Data collection methods	18a	Plans for assessment and collection of outcome, baseline, and other trial data, including any related processes to promote data quality (eg, duplicate measurements, training of assessors) and a description of study instruments (eg, questionnaires, laboratory tests) along with their reliability and validity, if known. Reference to where data collection forms can be found, if not in the protocol	9-10

For peer review only - http://bmjopen.bmj.com/site/about/guidelines.xhtml

	18b	Plans to promote participant retention and complete follow-up, including list of any outcome data to be collected for participants who discontinue or deviate from intervention protocols	8
Data management	19	Plans for data entry, coding, security, and storage, including any related processes to promote data quality (eg, double data entry; range checks for data values). Reference to where details of data management procedures can be found, if not in the protocol	13
Statistical methods	20a	Statistical methods for analysing primary and secondary outcomes. Reference to where other details of the statistical analysis plan can be found, if not in the protocol	11-12
	20b	Methods for any additional analyses (eg, subgroup and adjusted analyses)	12
	20c	Definition of analysis population relating to protocol non-adherence (eg, as randomised analysis), and any statistical methods to handle missing data (eg, multiple imputation)	12
**Methods: Monitoring**			
Data monitoring	21a	Composition of data monitoring committee (DMC); summary of its role and reporting structure; statement of whether it is independent from the sponsor and competing interests; and reference to where further details about its charter can be found, if not in the protocol. Alternatively, an explanation of why a DMC is not needed	12
	21b	Description of any interim analyses and stopping guidelines, including who will have access to these interim results and make the final decision to terminate the trial	N/A
Harms	22	Plans for collecting, assessing, reporting, and managing solicited and spontaneously reported adverse events and other unintended effects of trial interventions or trial conduct	10
Auditing	23	Frequency and procedures for auditing trial conduct, if any, and whether the process will be independent from investigators and the sponsor	N/A
**Ethics and dissemination**			
Research ethics approval	24	Plans for seeking research ethics committee/institutional review board (REC/IRB) approval	4

Protocol amendments	25	Plans for communicating important protocol modifications (eg, changes to eligibility criteria, outcomes, analyses) to relevant parties (eg, investigators, REC/IRBs, trial participants, trial registries, journals, regulators)	14
Consent or assent	26a	Who will obtain informed consent or assent from potential trial participants or authorised surrogates, and how (see Item 32)	5
	26b	Additional consent provisions for collection and use of participant data and biological specimens in ancillary studies, if applicable	N/A
Confidentiality	27	How personal information about potential and enrolled participants will be collected, shared, and maintained in order to protect confidentiality before, during, and after the trial	12
Declaration of interests	28	Financial and other competing interests for principal investigators for the overall trial and each study site	13
Access to data	29	Statement of who will have access to the final trial dataset, and disclosure of contractual agreements that limit such access for investigators	12
Ancillary and post-trial care	30	Provisions, if any, for ancillary and post-trial care, and for compensation to those who suffer harm from trial participation	9-10
Dissemination policy	31a	Plans for investigators and sponsor to communicate trial results to participants, healthcare professionals, the public, and other relevant groups (eg, via publication, reporting in results databases, or other data sharing arrangements), including any publication restrictions	13
	31b	Authorship eligibility guidelines and any intended use of professional writers	13
	31c	Plans, if any, for granting public access to the full protocol, participant-level dataset, and statistical code	N/A

Appendices

For peer review only - http://bmjopen.bmj.com/site/about/guidelines.xhtml

Informed consent materials	32	Model consent form and other related documentation given to participants and authorised surrogates	Available in Swedish upon request. Could be translated upon reasonable request.
Biological specimens	33	Plans for collection, laboratory evaluation, and storage of biological specimens for genetic or molecular analysis in the current trial and for future use in ancillary studies, if applicable	N/A

*It is strongly recommended that this checklist be read in conjunction with the SPIRIT 2013 Explanation & Elaboration for important clarification on the items. Amendments to the protocol should be tracked and dated. The SPIRIT checklist is copyrighted by the SPIRIT Group under the Creative Commons "Attribution-NonCommercial-NoDerivs 3.0 Unported" license.

For peer review only
